# Comprehensive tumour‐immune profiling reveals TREM2^+^ tumour‐associated macrophages facilitating lymph node metastasis in head and neck squamous cell carcinoma

**DOI:** 10.1002/ctm2.70604

**Published:** 2026-01-30

**Authors:** Zhuokai Wu, Chixing Cheng, Zhaoxin Li, Minyi Ren, Hongxi Cao, Weijie Huang, Jun Wang, Lixian Wu, Tingyi Lee, Sien Zhang, Hanhao Zheng, Yixi Wang

**Affiliations:** ^1^ Hospital of Stomatology, Guanghua School of Stomatology, Guangdong Provincial Key Laboratory of Stomatology, Sun Yat‐sen University Guangzhou China; ^2^ Department of Urology The Fifth Affiliated Hospital, Sun Yat‐sen University Zhuhai China; ^3^ Guangdong Provincial Key Laboratory of Malignant Tumor Epigenetics and Gene Regulation, Sun Yat‐sen Memorial Hospital, Sun Yat‐sen University Guangzhou China; ^4^ State Key Laboratory of Oncology in South China, Sun Yat‐sen University Cancer Center, Sun Yat‐sen University Guangzhou China; ^5^ Department of Urology Sun Yat‐sen Memorial Hospital, Sun Yat‐sen University Guangzhou China

**Keywords:** CD8^+^ Tex, HNSCC, LN metastasis, scRNA‐seq, TIME, TREM2^+^ TAMs

## Abstract

**Background:**

Lymph node (LN) metastasis is a well‐established independent prognostic factor in head and neck squamous cell carcinoma (HNSCC). Formation of suppressive tumour immune microenvironment (TIME) is a major contributor to tumour immune evasion and metastasis. However, the TIME landscape underlying LN‐metastatic HNSCC remains poorly elucidated.

**Methods:**

A total of 688 866 single‐cell transcriptomes across 212 HNSCC samples were integrated. Comprehensive bioinformatic analyses on single‐cell RNA sequencing and microarray datasets revealed a TREM2^+^ tumour‐associated macrophage (TAM) cluster associated with LN metastasis. The functional role of TREM2^+^ TAMs was investigated through multiplex immunohistochemistry (mIHC) staining in clinical HNSCC cohort and in vitro co‐culture experiments. Furthermore, machine learning algorithms were employed to construct a prognostic model for HNSCC.

**Results:**

Integrative single‐cell analysis revealed the immunosuppressive TIME of LN‐metastatic HNSCC, characterised by high infiltration of exhausted CD8^+^ T cells (CD8^+^ Tex). We identified a specific TREM2^+^ TAM cluster that was strongly associated with CD8^+^ Tex infiltration and LN metastasis. In vitro experiment confirmed that TREM2^+^ TAMs promoted CD8^+^ T cell exhaustion. Mechanistically, TREM2^+^ TAMs exhibited a terminally differentiated phenotype driven by ETV5, and secreted SPP1 to interact with CD44 on CD8^+^ T cells, thus upregulating BHLHE40 to promote CD8^+^ Tex formation. Clinically, a prognostic model based on TREM2^+^ TAM signature genes was trained to independently predict HNSCC outcomes.

**Conclusions:**

This study delineates the mechanism that TREM2^+^ TAMs promote LN metastasis in HNSCC by facilitating CD8^+^ T cells exhaustion via SPP1–CD44–BHLHE40 axis, proposing TREM2^+^ TAMs as potential therapeutic target for HNSCC.

## Graphical abstract

(1) TREM2^+^ TAMs promote CD8^+^ T cell exhaustion by upregulating BHLHE40 through the SPP1–CD44 axis, contributing to a suppressive tumour immune microenvironment in HNSCC. (2) CD8^+^ Tex induced by TREM2^+^ TAMs facilitates immune escape and promotes tumour cell metastasis via the lymphangion.

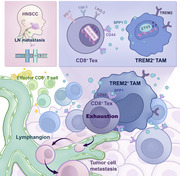

## 1 BACKGROUND

Head and neck squamous cell carcinoma (HNSCC) ranks as the sixth most common cancer globally, arising from the laryngeal, pharyngeal, or oral mucosal epithelium, and causes approximately 350 000 deaths annually.^1^ Despite advances in multimodal therapies, the overall 5‐year survival rate of patients with HNSCC remains around 50%.^2^ Among various prognostic factors,^3^ lymph node (LN) metastasis is the predominant metastatic manner and an independent prognostic factor for HNSCC.^4^ The majority of HNSCC patients present with regional LN metastasis at initial diagnosis.^5^ The 5‐year survival rate of HNSCC patients decreases progressively with increasing LN metastasis classification (N stage).^6^, ^7^ Notably, the survival rate for N2c (≥1 contralateral or bilateral LN ≤6 cm, without extranodal extension) classification or higher is less than 40%.^8^ Therefore, elucidating the molecular mechanisms driving LN metastasis is essential for improving the clinical outcomes of HNSCC patients.

The tumour immune microenvironment (TIME) plays a critical role in tumour progression and the response to immune therapy.^9^, ^10^ The suppressive TIME is a major contributor to tumour immune evasion and metastasis.^11^ Emerging evidence has confirmed that a suppressive TIME promotes LN metastasis by facilitating the formation of a metastatic niche and enhancing the colonisation and dissemination of circulating tumour cells.^12^, ^13^ Alteration towards immunosuppressive TIME is characterised by the abnormal infiltration of specific cellular components, including exhausted T cells (Tex), tumour‐associated macrophages (TAMs), myeloid‐derived suppressor cells (MDSCs), and cancer‐associated fibroblasts (CAFs).^14^, ^15^, ^16^, ^17^ Moreover, intercellular crosstalk plays a central role in regulating immune cell polarisation and biological behaviours, thereby contributing to the establishment of a suppressive TIME. However, the dynamic signature of the immune landscape underlying HNSCC LN metastasis remains insufficiently characterised.

**FIGURE 1:**
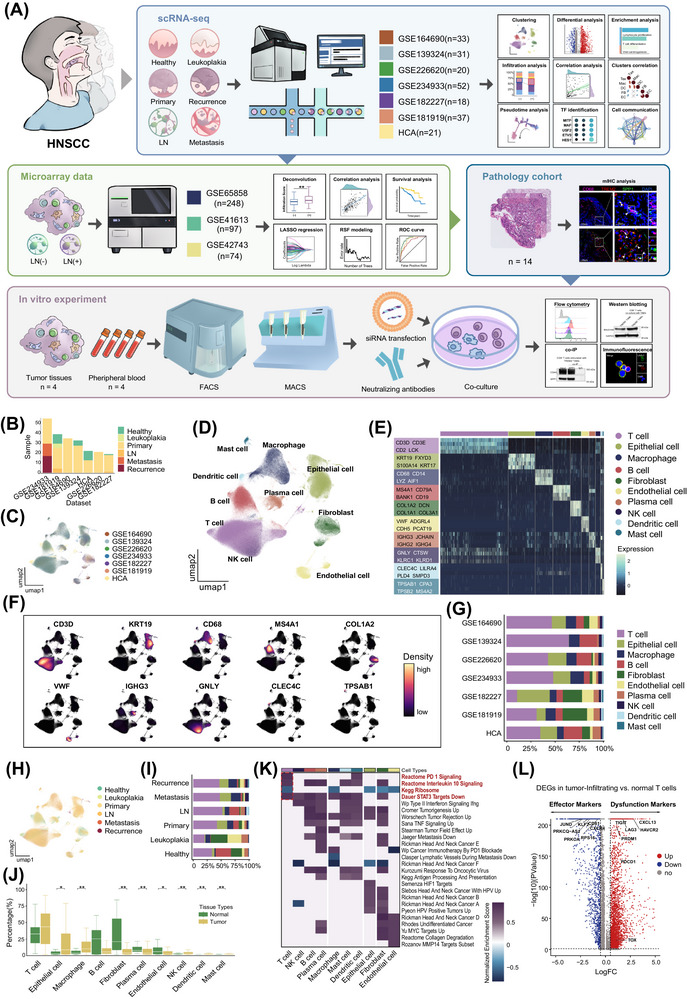
A single‐cell atlas of HNSCC. (A) Schematic workflow illustrating the study design and analytical approaches. (B) Bar plot showing the distribution of tissue types across each dataset. (C) UMAP plot displaying clustering result coloured by batches. (D) UMAP plot showing single‐cells coloured by cell types. (E) Heatmap depicting the top DEGs for each cell type. (F) UMAP plot visualising the expression density of feature genes for each cell type. (G) Bar plot representing the composition of cell types within each dataset. (H) UMAP plot showing single cells coloured by tissue types. (I) Bar plot illustrating the proportions of cell types across tissue types. (J) Comparison of cell infiltration percentages between normal and tumour tissues, ^*^ *p* < .05; ^**^ *p* < .01 by Mann–Whitney *U* test. (K) Heatmap of GSEA results based on DEGs between tumour and normal groups. (L) Volcano plot depicting DEGs in T cells between tumour and normal groups. DEGs, differentially expressed genes; GSEA, gene set enrichment analysis; HNSCC, head and neck squamous cell carcinoma; UMAP, uniform manifold approximation and projection.

The advent of high‐resolution single‐cell RNA sequencing (scRNA‐seq) has enabled investigation into heterogeneity among cell populations at the single‐cell level.^18^ However, due to the high cost of sequencing, previous studies on HNSCC TIME have been limited by the quantity of transcriptomic data, hindering comprehensive landscape mapping and precise subset identification.^19^ In the present study, to gain deeper insights into the TIME in HNSCC, we integrated 688 866 single cells from 212 samples across seven independent datasets and characterised the immune landscape of LN metastatic HNSCC at an unprecedented scale. By integrating scRNA‐seq datasets with microarray datasets, we identified a specific TREM2^+^ TAM cluster that was positively correlated with LN metastasis in HNSCC. Subsequent multiplex immunohistochemistry (mIHC) staining and co‐culture experiment revealed the mechanism that TREM2^+^ TAMs promote LN metastasis in HNSCC by facilitating CD8^+^ T cell exhaustion via SPP1–CD44–BHLHE40 axis. Clinically, a prognostic model based on TREM2^+^ TAMs further revealed its independent prognostic value. Our study demonstrated a previously unexplored dimension in the TIME of HNSCC, identifying TREM2^+^ TAMs as a potential therapeutic target for suppressing LN metastasis in HNSCC patients (Figure 1A).

## 2 METHODS AND MATERIALS

### 2.1 Data source

The scRNA‐seq data were collected from the Gene Expression Omnibus (GEO) database under accession codes GSE164690,^20^ GSE139324,^21^ GSE226620,^22^ GSE234933,^23^ GSE182227,^24^ GSE181919,^25^ and the Human Cell Atlas (HCA) database available at CZ CELLxGENE.^26^ We incorporated tissues from healthy donors, leukoplakia patients, primary tumour, metastatic LNs, distant metastatic lesions, and recurrent tumours, with peripheral blood mononuclear cells (PBMCs) sequencing data excluded to enable comprehensive analysis across disease stages on solid tissue. The microarray data were collected from the GEO database under accession codes GSE65858,^27^ GSE41613,^28^ and GSE42743.^28^ We selected treatment‐naïve primary tumour samples that contained TNM stage or LN metastasis information, and included only datasets that provided overall survival (OS) information.

### 2.2 Functional enrichment analyses

Gene Ontology (GO) and Kyoto Encyclopedia of Genes and Genomes (KEGG) pathway enrichment analyses were performed on the differentially expressed genes (DEGs) of each subset using the clusterProfiler and org.Hs.eg.db R packages.^29^ In addition, Gene Set Enrichment Analysis (GSEA) was conducted to assess the alteration of specific pathways between different groups or subsets.

### 2.3 Pseudotime analysis

To infer cellular differentiation trajectories, we applied multiple trajectory inference methods, including Diffusion Map,^30^ Monocle2,^31^ Monocle3,^32^ Slingshot,^33^ scTour,^34^ and CytoTRACE2.^35^ We employed Diffusion Map as the primary dimensionality reduction method using the destiny R package. Monocle2 was used for trajectories inference and gene‐level analyses by the Monocle R package.^31^ Monocle3 was implemented based on a principal component analysis (PCA) and Diffusion Map‐derived embedding via the *Monocle3* R packages.^32^ Slingshot estimated pseudotime by fitting principal curves over a minimum spanning tree (MST) on Diffusion Map coordinates, implemented via the slingshot R package. scTour captures dynamic transcriptional programs during differentiation using the sctour package in Python (version 3.12).^34^ We also estimated single‐cell developmental potency using the CytoTRACE2 R package, which learns binary gene‐expression programs associated with developmental potency categories.^35^ The directionality of trajectories was automatically inferred by scTour and CytoTRACE2, enabling the robust identification of roots for downstream pseudotime analyses.

### 2.4 Regulon analysis

To identify signature transcription factor (TF) within T cell and TAM subsets, we applied the SCENIC R package to infer gene regulatory networks by first identifying co‐expression modules and then linking them to candidate TFs to define potential regulons, each consisting of a TF and its predicted target genes.^36^ The Regulon Specificity Score (RSS) was calculated to assess the specificity of regulon activity across subsets. Area under the curve (AUC) scores were employed to assess the activity of each regulon in individual cells and were binarised to enable the depiction of activation status.

### 2.5 Cell–cell communication analysis

The CellChat R package was employed to investigate cell–cell communication among cells within the TIME.^37^ A CellChat object was constructed using the selected subsets as input, and all pre‐processing steps were performed using default parameters. To infer intercellular communication networks, a series of core functions were applied, including projectData, computeCommunProb, and filterCommunication (with a minimum threshold of 3 cells), followed by subsetCommunication and computeCommunProbPathway to identify significant ligand–receptor pairs and corresponding signalling pathways. The resulting communication networks were visualised using circle plots, bar plots, heatmaps, and chord diagrams, providing a comprehensive view of the intercellular signalling landscape across subsets.

### 2.6 Cell type deconvolution of microarray data

The MCP‐counter,^38^ CIBERSORTx,^39^ and EPIC^40^ algorithms were utilised in parallel to estimate the abundance of targeted subsets within the TIME based on microarray data. MCP‐counter quantifies cell population infiltration by applying predefined gene expression signatures identified in scRNA‐seq results. Similarly, CIBERSORTx and EPIC perform digital deconvolution of microarray profiles using reference gene matrices to infer the relative proportions of cell populations.

### 2.7 Patient samples

We obtained HNSCC samples from the Guanghua Hospital of Stomatology (Guangzhou, China), affiliated with Sun Yat‐sen University, with Institutional Review Board approval (KQEC‐2025‐065‐01). A cohort of 14 formalin‐fixed and paraffin‐embedded (FFPE) primary HNSCC tumour tissue slides and four pairs of freshly resected tumours and peripheral blood samples were collected, each derived from a distinct patient who met the following criteria: histologically confirmed operable HNSCC, no prior antitumour treatment, and neck LN dissection with clearly defined TNM staging.

### 2.8 mIHC and immunofluorescence staining analysis

For mIHC staining of tumour tissues, serial FFPE primary HNSCC tumour sections were used to assess the spatial distribution and relative positioning of subsets. Sections were dewaxed, rehydrated, and subjected to 15 min of antigen retrieval in ethylene diamine tetraacetic acid (EDTA) buffer at high temperature. The sections were subsequently blocked with goat serum for 30 min. And then sections were incubated with primary antibodies overnight at 4°C, followed by incubation with secondary antibodies and Opal tyramide signal amplification (TSA) dyes. Markers showing weak positivity were labelled with relatively strong dyes. The primary antibodies used included CD8a (Proteintech, 66868‐1‐Ig,), TIM‐3 (CST, 45208S), LAG‐3 (Abcam, ab209236), CD68 (Proteintech, 65515‐1‐RR), TREM2 (Abcam, ab318262), SPP1 (Proteintech, 83341‐1‐RR) and CD44 (Abcam, ab316123). The TSA dyes used were PPD480, PPD570, PPD620 and PPD690. The above process was repeated three times. 4′,6 ‐ diamidino ‐ 2 – phenylindole (DAPI) was then used to stain the nuclei. Sections were imaged under a digital slide scanner (Pannoramic MIDI, 3DHISTECH).

For immunofluorescence staining of cells, cells cultured on confocal dishes were fixed with 4% paraformaldehyde and subsequently blocked with goat serum for 1 h at room temperature to prevent nonspecific binding. The samples were then incubated overnight at 4°C with the indicated primary antibodies mentioned above, followed by incubation with fluorescently labelled secondary antibodies for 1 h at room temperature. DAPI was then used to stain the nuclei for 15 min at room temperature. Cells were imaged using a laser‐scanning confocal microscope (Olympus FV3000).

### 2.9 Cell isolation and culture

Cells were collected from paired tumour tissues and peripheral blood samples of four primary HNSCC patients. Freshly excised tumour tissues were processed using the Tumour Dissociation Kit (Miltenyi) following the manufacturer's instructions. Briefly, fibrotic and necrotic regions were removed, and the remaining tumour tissue was minced into small fragments. The fragments were digested with an enzyme mixture containing enzyme H (200 µL), enzyme R (100 µL), and enzyme A (25 µL) for 30 min at 37°C. Residual tissue pieces were subjected to the same treatment, and all resulting suspensions were combined. The cell suspension was filtered through a MACS SmartStrainer (70 µm), collected into a 50 mL tube, and centrifuged at 400 × *g* for 10 min to obtain a single‐cell pellet, which was resuspended in PBS. Peripheral blood samples were processed using density gradient centrifugation to isolate PBMCs. Fluorescence‐Activated Cell Sorting (FACS) was employed to purify live TREM2^+^ TAMs and TREM2^−^ TAMs from tumour‐derived cells, and Magnetic‐Activated Cell Sorting (MACS) was applied to purify CD8^+^ T cells from PBMCs via Human CD8 Positive Selection Kit 1 (STEMCELL). TAMs were cultured in RPMI 1640 medium (Gibco, Thermo Scientific) supplemented with 10 ng/mL M‐CSF (Yisheng Biotech) and HNSCC tissue lysate supernatant for 6 days. CD8^+^ T cells were activated with 20 ng/mL recombinant IL‐2 (PeproTech, Thermo Scientific) and 5 µg/mL αCD3/CD28 (STEMCELL) for 24 h. TAMs and CD8^+^ T cells were then co‐cultured in a 6‐well Transwell system (Corning,.4 µm pore size). CD8^+^ T cells were seeded in the lower chamber, and TAMs were plated in the upper chamber at a ratio of 1:1 in RPMI‐1640 medium supplemented with 10% FBS. The co‐culture was maintained at 37°C in a humidified 5% CO2 incubator for 48 h. After co‐culture, CD8^+^ T cells were collected from the lower chamber by gentle pipetting and centrifugation, TAMs were harvested by Pancreatin (Gibco, Thermo Fisher Scientific) digestion. In selected assays, 10 × 10^4^ CD8^+^ T cells with or without specific gene knockdown were seeded in flat‐bottom 96‐well culture plates and were activated with 20 ng/mL recombinant IL‐2 and 5 µg/mL αCD3/CD28 for 24 h. Then, CD8^+^ T cells were incubated with recombinant His‐tagged SPP1 (UABIOSCIENCE) with a final concentration of 20 nM for 48 h at 37°C in a humidified 5% CO2 incubator. After treatment, CD8^+^ T cells were harvested for subsequent experiments.

### 2.10 Cell transfection and blocking of target proteins

For cell transfection, activated CD8^+^ T cells were treated with siRNAs targeting BHLHE40 (si‐BHLHE40, IGE Bio) or CD44 (si‐CD44, IGE Bio) using a Bio‐Rad Gene Pulser Xcell™ with a square‐wave pulse (300 V, 10 ms, 1 pulse). Cells were then transferred to 24‐well plates containing complete medium with 20ng/mL IL‐2 and cultured at 37°C.Total RNA extracted from CD8^+^ T cells was reverse‐transcribed into cDNA using the HiScript III Reverse Transcriptase Kit (Vazyme), and the resulting cDNA was used to evaluate transfection efficiency by quantitative real‐time PCR (qRT‐PCR) with the ChamQ Universal SYBR qPCR Master Mix (Vazyme).

For the blocking of target proteins, target cells were separately treated with neutralising antibodies against SPP1 or CD44 (MedChemExpress) before co‐culture. Neutralising antibodies were added to the culture medium at a final concentration of 10 µg/mL each. Antibodies were gently mixed into the culture medium and incubated at 37°C for 2 h. A co‐culture experiment was conducted in the same medium without washing away antibodies.

### 2.11 FACS of TAMs and flow cytometry

For isolation of TREM2^+^ TAMs from tumour‐derived single‐cell suspensions, FACS was performed using a flow cytometer (Beckman MoFlo EOs). Immunocytes were first gated based on CD45 expression (BD Biosciences, 555482). TAMs were identified as CD11b^+^ cells (BD Biosciences, 552850). Within this population, the TREM2^+^ fraction was collected as TREM2^+^ TAMs by targeting TREM2 (R&D Systems, FAB17291P), whereas the remaining CD11b^+^ cells were designated as TREM2^−^ TAMs.

Flow cytometry was performed to analyse CD8^+^ T cell exhaustion and SPP1 expression in TREM2^+^ TAMs. CD8^+^ T cells were collected from the suspended fraction of the co‐culture medium in the co‐culture assay. Cells were processed into single‐cell suspensions, stained with a live/dead dye (BD Biosciences), and subsequently incubated with antibodies against specific surface markers for 20 min at room temperature. The following antibodies were used: CD8 (BioLegend, 344704), LAG‐3 (BD Biosciences, 565716), PD‐1 (BD Biosciences, 563245), and TIM‐3 (BD Biosciences, 565562) for CD8^+^ T cells; TREM2 (R&D Systems, FAB17291P) and SPP1 (eBioscience, 3002684) for TREM2^+^ TAMs. For intracellular protein detection, cells were fixed and permeabilised using the Flow Intracellular Fixation Rupture Buffer (eBioscience) before antibody staining. After staining, the expression levels of the indicated markers were measured using a FACScan flow cytometer (BD FACSCelesta; Beckman Coulter).

### 2.12 Prognostic model construction

A publicly available microarray dataset containing 248 samples (GSE65858) was used as the training set. A primary gene set comprising 1049 genes derived from scRNA‐seq analysis was used as the input feature set. In the first step, univariate Cox proportional hazards regression was performed using the survival R package to identify genes significantly associated with OS (*p* < .05). To enhance model interpretability and robustness, we subsequently applied Least Absolute Shrinkage and Selection Operator (LASSO) regression with ten‐fold cross‐validation using the glmnet R package (*alpha* = 1, *nlambda* = 100).^41^ The regularisation parameter was selected on the basis of the *λ.1se* criterion to obtain a more parsimonious predictive model by retaining the most informative genes. Following this, we employed a Random Survival Forest (RSF) algorithm using the randomForestSRC R package (ntree = 1000, nodesize = 10) to further refine the model.^42^ Only genes with positive importance values were retained for downstream modelling. These genes were then used to construct the final prognostic model, which was preliminarily evaluated using the concordance index (C‐index) through the R package survcomp to assess its predictive performance on the training dataset itself.^43^

### 2.13 Statistical analysis

Statistical analysis was performed with R (version 4.41) and GraphPad Prism.^44^ All experiments were conducted in three independent biological replicates; specific analytical methods are detailed in the corresponding subsections of the methods section. Two‐way ANOVA and Mann–Whitney *U* tests were applied to evaluate statistical significance between groups. Pearson's correlation analysis was used to assess the association between two variables. Survival curves were compared using log‐rank test. A *p*‐value of less than.05 was considered statistically significant, ^*^ *p* < .05, ^**^ *p* < .01.

## 3 RESULTS

### 3.1 Mapping the cellular landscape of HNSCC with a large‐scale atlas

To investigate the heterogeneity of the TIME in HNSCC, we first collected single‐cell transcriptome profiles from seven datasets (GSE164690, GSE139324, GSE226620, GSE234933, GSE182227, and GSE181919 from the GEO database, and a dataset from the HCA database), resulting in a total of 212 samples. All samples were classified into six tissue types representing the progression of HNSCC: healthy tissue (*n* = 24), leukoplakia (*n* = 4), primary tumour (*n* = 151), metastatic LN (*n* = 5), distant metastasis (*n* = 12), and recurrent tumour (*n* = 16) (Figure 1A,B). Strict quality control was performed individually for each dataset on the basis of violin plot distributions (Figure S1). After integration and batch effect removal, a total of 688 866 high‐quality cells were retained with a well‐mixed representation across datasets (Figure 1C,G). Using a shared nearest neighbour (SNN) graph‐based approach, 20 primary clusters were identified (Figure S2A). These clusters were further classified into ten major cell types on the basis of the expression of canonical marker genes (Figures 1D–F, S2B): epithelial cells (marked by KRT19 and S100A14), T cells (marked by CD2 and CD3), B cells (marked by MS4A1 and CD79A), plasma cells (marked by IGHG3 and JCHAIN), NK cells (marked by GNLY and KLRD1), macrophages (marked by CD68 and CD14), dendritic cells (marked by CLEC4C and LILRA4), mast cells (marked by TPSAB1 and TPSB2), fibroblasts (marked by COL1A2 and DCN) and endothelial cells (marked by VWF and CDH5). Further detection of subsets was performed to reveal the heterogeneity of the TIME (Figure S2C–E).

To reveal the heterogeneity of the immune landscape across different stages of HNSCC progression, we analysed the distribution of various cell types and found that the compositions varied across six stages (Figure 1H,I). Compared to healthy tissues, leukoplakia samples exhibited a marked increase in fibroblast and endothelial cell infiltration. In contrast, tumour tissues, including primary tumours, distant metastases, and recurrent tumours, showed a significant increase in epithelial cell proportion, as well as an increased presence of myeloid cells, including dendritic cells, macrophages, and mast cells. Moreover, metastatic LN was characterised by an increased infiltration of lymphoid cells, particularly T cells and B‐lineage cells (B cells and plasma cells) (Figure 1I). To characterise immune microenvironmental shifts in HNSCC, we dichotomised the six tissue types into normal (healthy tissue and leukoplakia) and tumour (primary tumour, metastatic LN, distant metastasis, and recurrent tumour) groups. A clear difference emerged: the tumour group showed a significant increase in epithelial cell proportion, accompanied by marked decreases in stromal cells (fibroblasts and endothelial cells) and B‐lineage cells. Notably, macrophage infiltration was significantly higher in the tumour group. Although the increase in T cell infiltration did not reach statistical significance (*p* > .05), its upper bound was much higher in tumour tissues (Figure 1J). GSEA functional enrichment comparing tumour and normal groups revealed that most immune cells exhibited immune activation in tumour. In contrast, T cells showed signs of immunosuppression and reduced proliferation, characterised by PD‐1 and IL‐10 signalling upregulation, together with decreased ribosomal activity and attenuated STAT3‐mediated transcriptional activity, suggesting T cell dysfunction in tumour progression (Figure 1K). Further analysis of DEGs demonstrated that T cells in tumour tissues highly expressed dysfunction markers including LAG3 (encoding lymphocyte activation gene 3, LAG‐3), PDCD1 (encoding programmed cell death protein 1, PD‐1), HAVCR2 (encoding hepatitis A virus cellular receptor 2, TIM‐3), and TOX (encoding thymocyte selection–associated high mobility group box protein, TOX) (Figure 1L). Taken together, we profiled a total of 688 866 cells across six tissue types, constructing a comprehensive cellular landscape of HNSCC. The analysis revealed a profoundly immunosuppressive microenvironment, with a particular emphasis on T cell dysfunction.

### 3.2 CD8^+^ Tex infiltration positively correlates with LN metastasis in HNSCC

**FIGURE 2:**
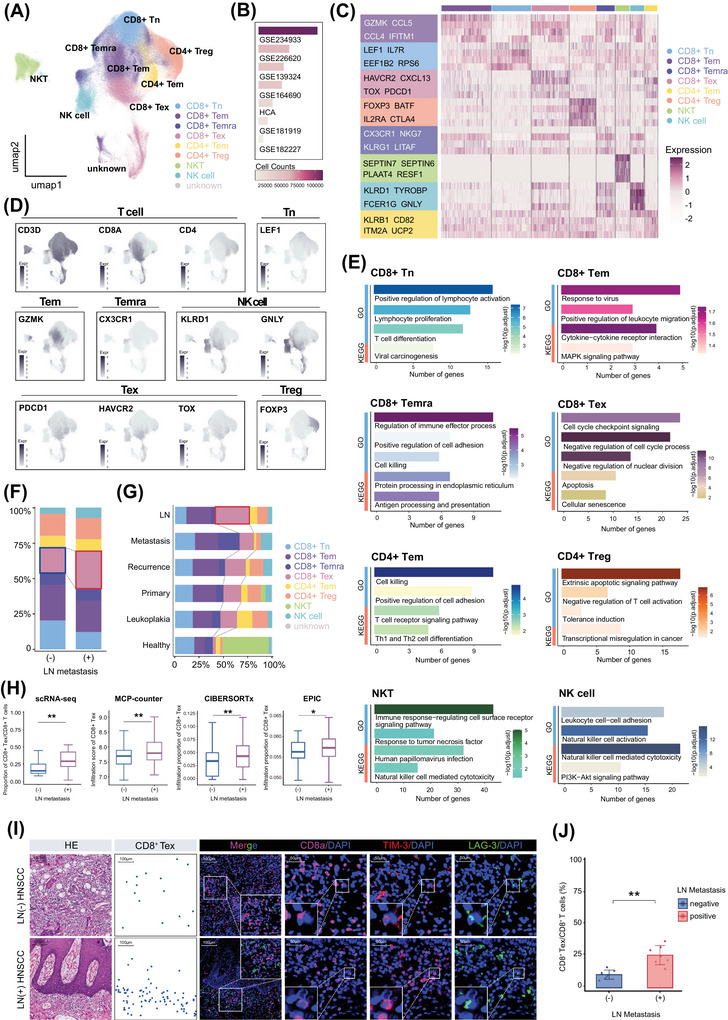
CD8^+^ Tex infiltration increases in LN metastatic HNSCC. (A) UMAP plot of T cell subsets coloured by the identified subsets. (B) Bar plot showing the number of cells contributed by each dataset. (C) Heatmap displaying the top DEGs of T cell subsets. (D) UMAP plot visualising the expression of feature genes of each T cell subset. (E) GO and KEGG functional enrichment analysis of each T cell subset. (F) Bar plot showing the proportion of T cell subsets in LN metastasis‐positive and ‐negative tumour tissues, highlighting CD8^+^ Tex. (G) Bar plot showing the composition of T cell subsets in each tissue type, highlighting CD8^+^ Tex in metastatic LN. (H) Infiltration analyses on scRNA‐seq datasets, and by MCP‐counter, CIBERSORTx and EPIC deconvolution analyses on microarray dataset showing abundance of CD8^+^ Tex in LN metastasis‐positive and ‐negative tumour tissues, ^**^ *p* < .01 by Mann–Whitney *U* test. (I) Representative mIHC images of CD8^+^ Tex infiltration in LN metastasis‐positive and ‐negative tumour tissue, using CD8a, TIM‐3, and LAG‐3 as surface markers. Scale bar: 100 µm; inset scale bar: 50 µm. (J) Quantification of CD8^+^ Tex proportion among CD8^+^ T cells across LN metastasis‐positive (*n* = 7) and ‐negative (*n* = 7) tumour tissues by mIHC. Error bars show the mean ± SD, ^**^ *p* < .01 by Two‐way ANOVA test. ANOVA, Analysis of Variance; DEGs, differentially expressed genes; GO, Gene Ontology; HNSCC, head and neck squamous cell carcinoma; KEGG, Kyoto Encyclopedia of Genes and Genomes; LN, lymph node; mIHC, multiplex immunohistochemistry; scRNA‐seq, single‐cell RNA sequencing; SD, standard deviation; Tex, exhausted T cells; UMAP, uniform manifold approximation and projection.

T cells are considered central players in the TIME, mediating crucial antitumour immune responses.^45^ To assess the dynamic spectrum and functional heterogeneity of T cells in HNSCC, we performed re‐clustering and identified seven major T cell subsets for in‐depth analysis (Figures 2A, S3A). T cells across all datasets and tissue types were jointly analysed (Figures 2B,S3B,C). T cell subsets were characterised on the basis of their comprehensive transcriptomic profiles and canonical marker genes: naïve CD8^+^ T cells (CD8^+^ Tn, marked by LEF1 and IL7R), effector memory CD8^+^ T cells (CD8^+^ Tem, marked by GZMK and CCL5), terminally differentiated effector memory CD8^+^ T cells (CD8^+^ Temra, marked by CX3CR1 and KLRG1), exhausted CD8^+^ T cells (CD8^+^ Tex, marked by HAVCR2 and PDCD1), effector memory CD4^+^ T cells (CD4^+^ Tem, marked by KLRB1 and CD82), regulatory CD4^+^ T cells (CD4^+^ Treg, marked by FOXP3 and BATF), and natural killer T cells (NKT, marked by SEPTIN7 and SEPTIN6). Additionally, natural killer cells (NK cells, marked by KLRD1 and GNLY) were included during re‐clustering due to their transcriptional similarity to T cells (Figure 2C,D). GO and KEGG enrichment analyses revealed distinct functional profiles among T cell subsets (Figure 2E). CD8^+^ Tn exhibited active proliferative and differentiation potential, whereas CD8^+^/CD4^+^ Tem displayed enhanced effector functions, which were further amplified in CD8^+^ Temra. In addition, CD4^+^ Treg contributed to immune tolerance, while NKT cell demonstrated cytotoxic activity comparable to that of NK cells. Notably, CD8^+^ Tex were highly enriched in pathways related to cell cycle arrest and apoptosis, which was consistent with the immunosuppressive landscape observed in HNSCC (Figure 2E).

LN metastasis is considered a key driver of HNSCC progression and is strongly associated with poor OS.^6^, ^7^ To characterise the T cell subset associated with LN metastasis, we conducted a comparative analysis of the proportions of subsets in LN metastasis‐positive and ‐negative primary tumour tissues. The results revealed a significant increase in CD8^+^ Tex and a decrease in CD8^+^ Tn in LN metastasis‐positive group compared with LN metastasis‐negative group (Figures 2F, S3D). Meanwhile, we observed an increase in CD8^+^ Tex infiltration in the metastatic LN samples (Figures 2G, S3G,H). The distinctiveness of CD8^+^ Tex between LN metastasis‐positive and ‐negative groups was then statistically confirmed by cell counts per sample (Figure 2H). To validate our observations, a deconvolution analysis of microarray data from 248 samples was performed using MCP‐counter, CIBERSORTx and EPIC to quantify CD8^+^ Tex infiltration. Consistently, all methods confirmed that CD8^+^ Tex abundance was positively correlated with LN metastasis in HNSCC (Figure 2H). Moreover, by using the MiloR statistical framework, we found that CD8^+^ Tex were significantly enriched in LN metastasis‐positive group (Figure S3E,F). To further substantiate our findings on real patient tumour tissues, we performed mIHC staining. Specifically, CD8a, TIM‐3, and LAG‐3 were used as markers to identify CD8^+^ Tex. The results revealed that while the overall infiltration of CD8^+^ T cells was comparable between LN metastasis‐positive and ‐negative HNSCC samples, the proportion of CD8^+^ Tex within the CD8^+^ T cell population was significantly higher in tumours with LN metastasis (Figure 2I,J), supporting the strong association between elevated CD8^+^ Tex abundance and LN metastasis in HNSCC.

### 3.3 BHLHE40 is a key TF driving CD8^+^ Tex formation in HNSCC

**FIGURE 3:**
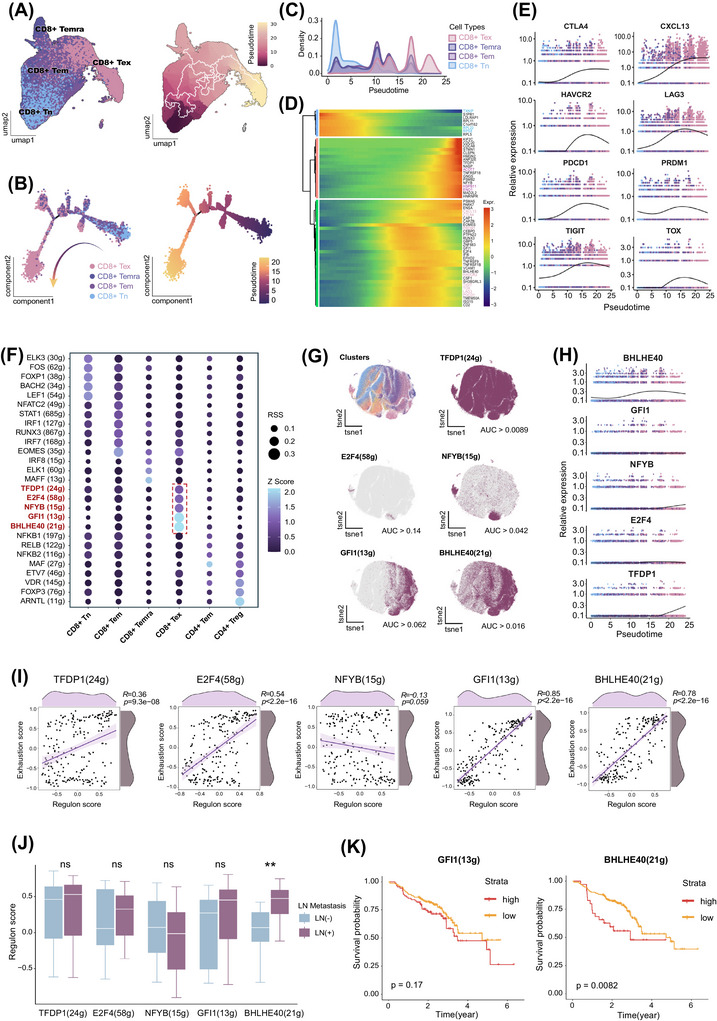
BHLHE40 plays a significant role in the differentiation of CD8^+^ Tex. (A) differentiation trajectory of CD8^+^ T cells coloured by subsets (left) and pseudotime (right) by Monocle3. (B) Differentiation trajectory of CD8^+^ T cells coloured by subsets (left) and pseudotime (right) by Monocle2. (C) Density distribution of CD8^+^ T cell subsets along pseudotime. (D) Heatmap of highly variable genes during CD8^+^ T cell differentiation pseudotime. (E) Expression dynamics of classical exhaustion markers along pseudotime. (F) Dot plot displaying the top TF regulons by RSSs of CD8^+^ T cell subsets. (G) t‐distributed Stochastic Neighbour Embedding (t‐SNE) reduction based on AUC scores of TF regulons. The top‐left panel shows cell subsets, the remaining panels highlight cells that activate each regulon (AUC > threshold). (H) Expression of CD8^+^ Tex‐specific TFs along pseudotime. (I) Correlation between activating score of CD8^+^ Tex‐specific TF regulons and exhaustion scores by Spearman correlation. (J) Comparison of activating score of CD8^+^ Tex‐specific TF regulons between LN metastasis‐positive and ‐negative samples. ns: not significant; ^**^ *p* < .01 by the Mann–Whitney U test. (K) Kaplan–Meier survival curves based on GFI1 and BHLHE40 regulon scores in HNSCC patients. The samples were stratified into high‐ and low‐score groups (optimal cutoff), using log‐rank test. AUC, Area under the curve; HNSCC, head and neck squamous cell carcinoma; LN, lymph node; ns, not significant; RSS, regulon specificity score; t‐SNE, t‐distributed Stochastic Neighbour Embedding; Tex, exhausted T cells; TF, transcription factor.

To investigate the developmental origin of CD8^+^ Tex, we first constructed the differentiation trajectory of CD8^+^ T cells in HNSCC. Monocle3 inferred two distinct terminal fates of CD8^+^ T cells: the first trajectory began with CD8^+^ Tn, progressed through CD8^+^ Tem, and terminated at CD8^+^ Temra; the second trajectory, which appeared to be more dominant, similarly started with CD8^+^ Tn, however, transitioned through CD8^+^ Tem and CD8^+^ Temra, and ultimately culminated in CD8^+^ Tex (Figure 3A). Complementary analysis by Monocle2 further underscored the linear differentiation trajectory ending in CD8^+^ Tex (Figure 3B,C), suggesting that CD8^+^ Tex represents the predominant terminal state of CD8^+^ T cells. DEG analysis along the differentiation trajectory revealed three major expression patterns. Genes such as TXNIP, RPL22 and RPS8 were highly expressed at the early stage, indicating T cell proliferation and activation. In the intermediate stage, classical exhaustion markers including PDCD1, HAVCR2, CTLA4, TOX and LAG3 reached peak expression and remained elevated thereafter (Figures 3D,E, S4A). In the late stage, genes involved in metabolic reprogramming, such as ACOT7, HSPB11 and ENO1, were upregulated, suggesting a metabolically adapted and sustained exhausted state (Figure 3D), indicating that CD8^+^ Tex represent a terminal exhaustion phenotype.

To characterise the core TF governing CD8^+^ Tex formation, SCENIC analysis was performed. This analysis identified five signature TF regulons with the highest specificity scores to CD8^+^ Tex: TFDP1, E2F4, NFYB, GFI1, and BHLHE40 (Figure 3F). First, we examined the distribution patterns of these TFs and found that NFYB, GFI1, and BHLHE40 exhibited distributions closely aligned with the localisation of CD8^+^ Tex (Figure 3G), suggesting their specific expression in CD8^+^ Tex. In addition, TFDP1, NFYB, and BHLHE40 were upregulated at the terminal stage of the differentiation trajectory, with BHLHE40 showing the most consistent dynamic pattern with exhaustion markers (Figure 3H). Among the five TFs, all except NFYB were significantly positively correlated with exhaustion marker expression scores, with GFI1 (*R* = .85) and BHLHE40 (*R* = .78) showing the strongest correlations (Figure 3I), suggesting their potential involvement in the exhaustion program. Notably, comparative analysis revealed that only BHLHE40 was significantly upregulated in LN metastasis‐positive group compared to LN metastasis‐negative group (Figure 3J). Clinically, survival analysis further revealed that high BHLHE40 expression was associated with reduced OS, whereas this trend was not statistically significant for other TFs (Figures 3K, S4B). Taken together, these findings suggest that BHLHE40 is the potential driver of CD8^+^ Tex formation and associated adverse clinical outcomes in HNSCC.

### 3.4 TREM2^+^ TAMs promote CD8^+^ T cell exhaustion through upregulating BHLHE40

**FIGURE 4:**
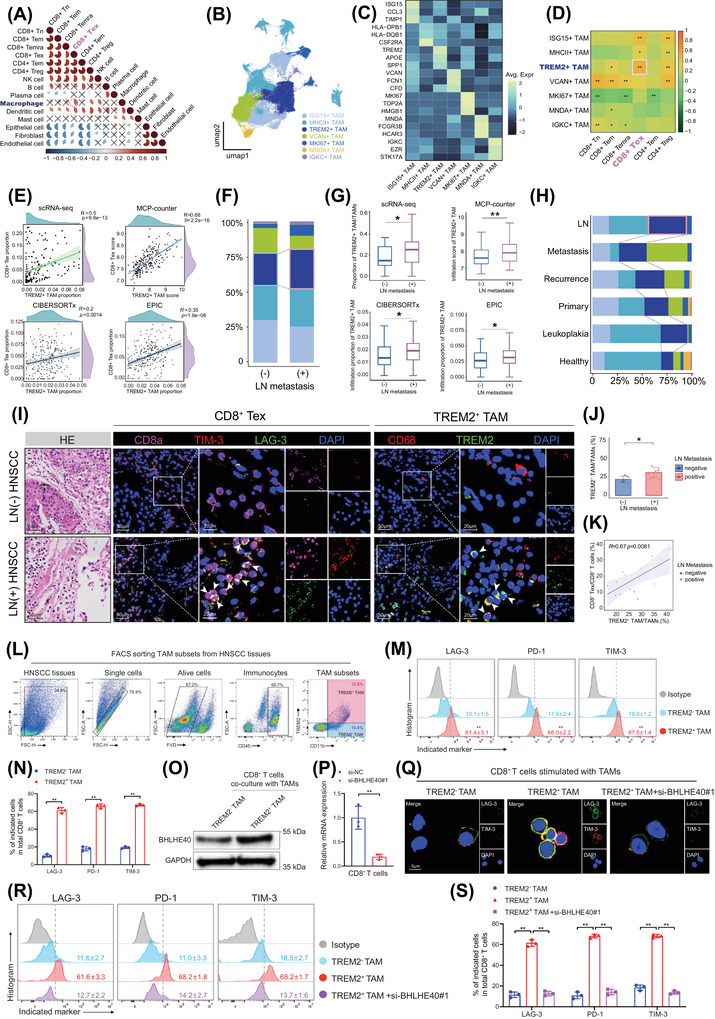
TREM2^+^ TAMs co‐infiltrate with CD8^+^ Tex. (A) Correlation matrix of infiltration proportions between cell types, crosses denote *p* > .05 by Spearman test. (B) UMAP visualisation of TAM subsets. (C) Heatmap displaying top DEGs across TAM subsets. (D) Correlation matrix of infiltration proportions among TAM and T cell subsets, ^*^ *p *< .05, ^**^ *p *< .01 by Spearman test. (E) Correlation of infiltration between TREM2^+^ TAMs and CD8^+^ Tex in scRNA‐seq and microarray datasets by Spearman test. (F) Proportions of TAM subsets in LN metastasis‐positive and ‐negative tumour tissues. (G) Infiltration analyses in scRNA‐seq and microarray datasets showing abundance of TREM2^+^ TAMs in LN metastasis‐positive and ‐negative tumour tissues, ^*^ *p* < .05, ^**^ *p *< .01 by Mann–Whitney U test. (H) Distribution of TAM subsets across tissue types. (I) Representative mIHC images showing co‐infiltration of CD8^+^ Tex and TREM2^+^ TAMs in LN metastasis‐positive and ‐negative tumour tissues. Scale bar, 50 µm; insets, 20 µm. (J) Proportion of TREM2^+^ TAMs among total TAMs in LN metastasis‐positive and ‐negative tumours in mIHC. Error bars show the mean ± SD, ^**^ *p* < .01 by Two‐way ANOVA test. (K) Correlation of infiltration levels between TREM2^+^ TAMs and CD8^+^ Tex in mIHC by Spearman test. (L) FACS of TAM subsets from HNSCC tissues. (M) and (N) Flow cytometric analysis of exhaustion markers on CD8^+^ T cells stimulated with TAM subsets. Error bars show the mean ± SD, ^**^ *p* < .01 by Two‐way ANOVA test. (O) Western blotting analysis of BHLHE40 expression in CD8+ T cells co‐cultured with TAM subsets. (P) qRT‐PCR result showing relative BHLHE40 mRNA expression in CD8^+^ T cells transfected with si‐NC or si‐BHLHE4. Error bars show the mean ± SD, ^**^ *p* < .01 by Two‐way ANOVA test. (Q) Immunofluorescence showing the expression of exhaustion markers on CD8^+^ T cells with or without BHLHE40 silencing co‐cultured with TAM subsets. Scale bar, 5 µm. (R) and (S) Flow cytometric analysis of exhaustion markers expression on CD8^+^ T cells with or without BHLHE40 silencing co‐cultured with TAM subsets. Error bars show the mean ± SD, ^**^ *p* < .01 by Two‐way ANOVA test. ANOVA, Analysis of Variance; DEGs, differentially expressed genes; FACS, Fluorescence‐Activated Cell Sorting; HNSCC, head and neck squamous cell carcinoma; LN, lymph node; mIHC, multiplex immunohistochemistry; mRNA, messenger RNA; qRT‐PCR, quantitative real‐time PCR; scRNA‐seq, single‐cell RNA sequencing; SD, standard deviation; si‐NC, small interfering RNA negative control; TAMs, tumour‐associated macrophages; Tex, exhausted T cells; UMAP, uniform manifold approximation and projection.

Crosstalk with interstitial cells in the TIME is a crucial factor driving the formation of CD8^+^ Tex.^46^ To delineate the cellular contributors to the exhaustion of CD8^+^ T cells, we first examined the correlations among T cell subsets and other components within the HNSCC TIME. The analysis revealed that TAMs exhibited a specific positive correlation with CD8^+^ Tex (Figure 4A). Given the well‐recognised heterogeneity of TAMs,^47^ we performed a re‐clustering on TAMs across datasets (Figure S5A). This analysis identified seven distinct TAM subsets on the basis of their comprehensive expression profiles: ISG15^+^ TAMs (marked by ISG15, CCL3 and TIMP1), MHCII^+^ TAMs (marked by MHC class II molecules and CSF2RA), TREM2^+^ TAMs (marked by TREM2, APOE and SPP1), VCAN^+^ TAMs (marked by VCAN, FCN1 and CFD), MKI67^+^ proliferating TAMs (marked by MKI67, TOP2A and HMGB1), MNDA^+^ TAMs (marked by MNDA, FCGR3B and HCAR3), and IGKC^+^ TAMs (marked by IGKC, EZR and STK17A) (Figures 4B,C, S5B). Intermixing of datasets and tissue types demonstrated successful integration of each subset (Figure S5C,D). Among all TAM subsets, correlation analysis revealed that TREM2^+^ TAMs exhibited the strongest association with CD8^+^ Tex, with a correlation coefficient of *R* = .50 (*p* < .01) (Figure 4D,E). Further validation on microarray data by deconvolution analyses revealed a consistent correlation between TREM2^+^ TAMs and CD8^+^ Tex (Figure 4E). More importantly, intergroup comparison among all TAM subsets demonstrated that although TREM2^+^ TAMs and ISG15^+^ TAMs both present strong correlation with CD8^+^ Tex, TREM2^+^ TAMs were markedly enriched in LN metastasis‐positive tumour tissues, which was then validated by both scRNA‐seq and microarray data, whereas ISG15^+^ TAMs were decreased in LN metastasis‐positive group and were therefore excluded from further analysis (Figures 4F,G, S5E). Moreover, we further evaluated the intergroup distribution differences by MiloR to confirm the elevation of TREM2^+^ TAMs in LN metastasis‐positive group (Figure S5F,G). Furthermore, a similar increase in TREM2^+^ TAM infiltration was also observed in metastatic LN, while their infiltration was minimal in healthy tissues (Figures 4H, S5H,I). Functionally, enrichment analysis revealed that TREM2^+^ TAMs from LN metastatic group significantly upregulated “Interactions with lymphoid cells” pathway compared with non‐metastatic group, supporting the correlation between TREM2^+^ TAMs–CD8^+^ Tex crosstalk and LN metastasis (Figure S6A,B). Molecularly, our analysis revealed a strong positive association between the proportion of TREM2^+^ TAMs and BHLHE40 expression in CD8^+^ Tex (Figure S6E). These findings suggested that TREM2^+^ TAMs may represent a potential subset promoting CD8^+^ T cell exhaustion by modulating their key TF.

To validate our findings in tumour tissues, we performed mIHC staining and detected a higher density of TREM2^+^ TAMs in LN metastasis‐positive tumour tissues (Figure 4I,J). Furthermore, TREM2^+^ TAMs exhibited a strong spatial association with CD8^+^ Tex, which was more pronounced in LN metastasis‐positive tumour tissues (Figure 4I,K). These findings supported the notion that TREM2^+^ TAMs may facilitate CD8^+^ T cell exhaustion and contribute to LN metastasis in HNSCC. To investigate whether TREM2^+^ TAMs induce CD8^+^ T cell exhaustion in vitro, TAM subsets were subjected to co‐culture with CD8^+^ T cells. The results showed that CD8^+^ T cells co‐cultured with TREM2^+^ TAMs exhibited markedly increased expression of exhaustion markers (LAG‐3, PD‐1 and TIM‐3) and BHLHE40 compared with those co‐cultured with TREM2^−^ TAMs (Figure 4L–O). Moreover, immunofluorescence staining and flow cytometric results consistently confirmed that silencing BHLHE40 in CD8^+^ T cells attenuated the ability of TREM2^+^ TAMs to upregulate exhaustion markers on CD8^+^ T cells (Figure 4P–S). Collectively, these findings demonstrate that TREM2^+^ TAMs promote CD8^+^ T cell exhaustion by enhancing BHLHE40 expression, thereby contributing to LN metastasis in HNSCC.

### 3.5 ETV5 governs the differentiation toward TREM2^+^ TAM phenotype

**FIGURE 5:**
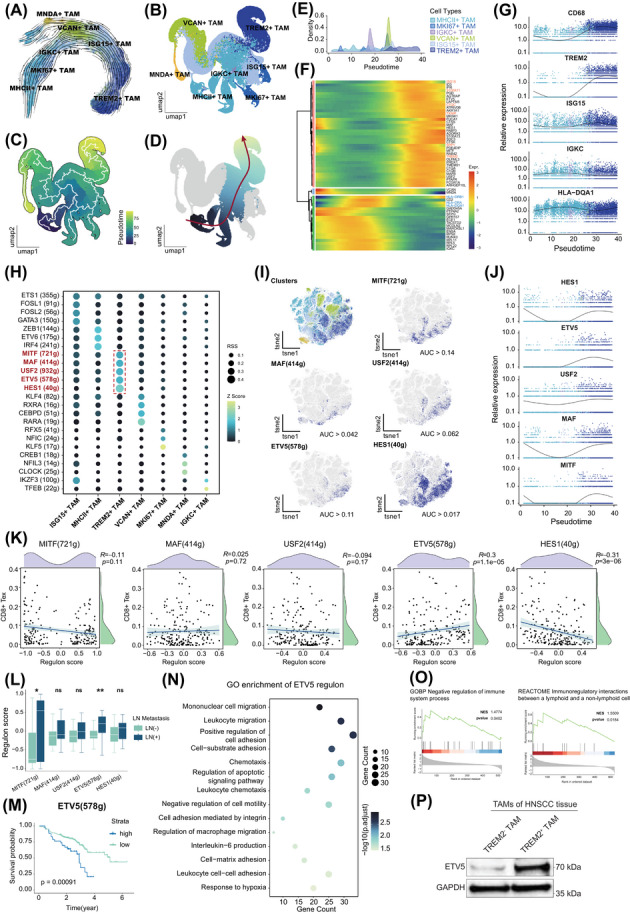
ETV5 contributes to the differentiation of TREM2^+^ TAMs. (A) Trajectory of TAM subsets by scTour. (B) TAM subsets on Diffusion Map, coloured by subsets. (C) Trajectory of TAM subsets by Monocle3 on Diffusion Map. (D) Differentiation lineage of TAMs by Slingshot on Diffusion Map, D–G, and J are based on Slingshot results. (E) Distribution of TAM subsets along pseudotime. (F) Heatmap showing differentiation‐related genes in TAMs. (G) Expression of main TAM subset markers along pseudotime. (H) Dot plot displaying the top TF regulons with highest RSSs of TAM subsets. (I) t‐SNE plot based on AUC scores of TF regulons. The top‐left panel shows cell subsets, the remaining panels highlight cells that activate each regulon (AUC > threshold). (J) Expression of TREM2^+^ TAM‐specific TFs along pseudotime. (K) Spearman correlation between the scores of TREM2^+^ TAM‐specific TF regulons and the proportion of CD8^+^ Tex. (L) Regulon scores of each TREM2^+^ TAM‐specific TF between LN metastasis‐positive and ‐negative groups, ns not significant, ^*^ *p* < .05, ^**^ *p* < .01 by Mann–Whitney *U* test. (M) Kaplan–Meier survival curves generated for ETV5 regulon scores from microarray datasets. The samples were divided into high‐ and low‐score groups (optimal cutoff), using the log‐rank test. (N) GO enrichment of the ETV5 regulon gene set, the top 14 pathways were shown. (O) GSEA results comparing ETV5 regulon gene set in TREM2^+^ TAMs and other TAM subsets. (P) Western blotting analysis of ETV5 expression in TAM subsets. AUC, Area under the curve; GO, Gene Ontology; GSEA, Gene Set Enrichment Analysis; LN, lymph node; ns, not significant; RSS, Regulon Specificity Score; scTour, single‐cell trajectory inference using optimal transport; TAMs, tumour‐associated macrophages; Tex, exhausted T cells; TF, transcription factor; t‐SNE, t‐distributed Stochastic Neighbour Embedding.

To investigate the developmental stage of TREM2^+^ TAMs, we applied multiple pseudotime inference algorithms. The trajectory generated by scTour revealed a primary path initiating from MHCII^+^ TAMs, progressing through intermediate states, and culminating in TREM2^+^ TAMs (Figure 5A). Moreover, CytoTRACE2 revealed a similar developmental potential index, confirming the lowest developmental potency of TREM2^+^ TAMs (Figure S6C,D). Monocle3 inferred the differentiation trajectory on the basis of Diffusion Map, defining TREM2^+^ TAMs as the dominant terminal state of the trajectory (Figure 5B,C). Subsequent analysis with Slingshot further clarified that a trajectory began with MHCII^+^ TAMs, passed through MKI67^+^ TAMs and IGKC^+^ TAMs, transitioned via ISG15^+^ TAMs, and finally ended with TREM2^+^ TAMs (Figure 5D,E). Gene expression dynamics along the trajectory revealed key regulatory changes: genes related to MHCII‐mediated antigen presentation progressively declined, suggesting a decrease in antigen‐presenting capability. In contrast, the upregulated expression of ISG15, S100A11, C1QC, TXNIP, SPP1, and TREM2 highlighted a shift toward an immunoregulatory phenotype in the late stage (Figure 5F,G). Together, these observations indicated that TREM2^+^ TAMs formed a terminally differentiated subset of TAMs.

To identify key TF involved in the formation of TREM2^+^ TAMs, SCENIC analysis was performed. This analysis identified MITF, MAF, USF2, ETV5, and HES1 as the top TF regulons specifically activated in TREM2^+^ TAMs (Figure 5H). Notably, the activity patterns of all five regulons showed strong spatial concordance with TREM2^+^ TAMs (Figure 5I). Furthermore, the expression dynamics of all five TFs closely mirrored that of TREM2 (Figure 5J), supporting their potential involvement in the differentiation of TREM2^+^ TAMs. To further pinpoint the TF most closely associated with CD8^+^ Tex, we evaluated the correlation between TF regulon scores and the infiltration level of CD8^+^ Tex, which elucidated that only ETV5 exhibited a significant positive correlation with CD8^+^ Tex infiltration (Figure 5K). Subsequent intergroup comparisons along with survival analysis on microarray data indicated that elevated ETV5 activity was specifically associated with both LN metastasis and increased mortality risk in HNSCC patients (Figures 5L,M, S6F). GO and GSEA enrichment analyses further demonstrated that ETV5 regulon activation was associated with pathways related to negative regulation of immune processes, and interaction with lymphoid cells (Figure 5N,O). More importantly, we then detected a high transcriptional activity of ETV5 in TREM2^+^ TAMs in vitro (Figure 5P). Taken together, these findings suggested that ETV5 activation may initiate the development of TREM2^+^ TAMs and strengthen their interaction with CD8^+^ Tex.

### 3.6 TREM2^+^ TAMs induce CD8^+^ Tex formation through the SPP1–CD44 axis

**FIGURE 6:**
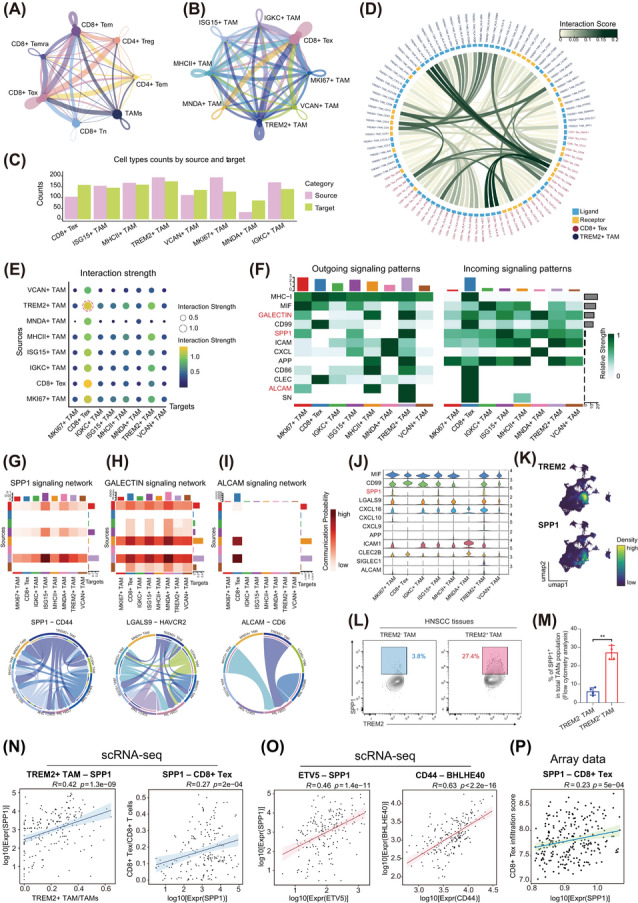
TREM2^+^ TAMs crosstalk with CD8^+^ Tex through SPP1–CD44 signalling pathway. (A) Circle plot depicting the crosstalk among TAMs and T cell subsets. Width indicates the weight of the interaction strength. (B) Circle plot depicting the crosstalk among the CD8^+^ Tex and TAM subsets. Width indicates the weight of the interaction strength. (C) Interaction frequency of each subset as senders (source) and receivers (target). (D) Chord diagram showing all the ligand–receptor interactions between TREM2^+^ TAMs and CD8^+^ Tex, coloured by interaction strength. (E) Interaction strength between pairwise cell subsets, coloured by strength score. (F) Heatmap illustrating pathways involved in the interaction between TREM2^+^ TAMs and CD8^+^ Tex, outgoing signalling (left), and incoming signalling (right), coloured by interaction strength. (G)–(I) Heatmap showing the interaction probability of signalling pathways among subsets (top), and chord diagram displaying the interaction strength of corresponding ligand–receptor pairs among subsets (bottom). (J) Violin plot showing the expression level of ligands in TAM subsets. (K) UMAP plot visualising the expression density of TREM2 and SPP1 in TAMs. (L) and (M) Flow cytometric analysis showing SPP1 expression in TAM subsets (*n* = 4). Error bars show the mean ± SD, ^**^ *p* < .01 by Two‐way ANOVA test. (N) Correlation between the expression level of SPP1 with the proportion of TREM2^+^ TAM/TAMs (left) and the proportion of CD8^+^ Tex/CD8^+^ T cells (right) across samples by Spearman test. (O) Correlations between the expression level of SPP1 with ETV5 (left) and BHLHE40 (right) across samples by Spearman test. (P) Correlations between the expression level of SPP1 with the proportion of CD8^+^ Tex in microarray dataset using MCP‐counter deconvolution by Spearman test. ANOVA, Analysis of Variance; SD, standard deviation; TAMs, tumour‐associated macrophages; Tex, exhausted T cells; UMAP, uniform manifold approximation and projection; ns, not significant.

To further investigate the mechanism by which TREM2^+^ TAMs induce CD8^+^ Tex, cell–cell communication analysis was performed using CellChat. First, analysis of the interactions between TAMs and T cell subsets revealed that CD8^+^ Tex received most signals from TAMs among T cells (Figure 6A), and specifically from TREM2^+^ TAMs (Figure 6B). Furthermore, quantification of interaction frequencies revealed that TREM2^+^ TAMs acted as the main signal sender, whereas CD8^+^ Tex functioned primarily as receivers (Figure 6C). A global chord diagram highlighting the interactions between TREM2^+^ TAMs and CD8^+^ Tex revealed their multilayered crosstalk (Figure 6D). Overall, TREM2^+^ TAMs ranked as the strongest contributors of signals toward CD8^+^ Tex among all the TAM subsets (Figure 6E), suggesting that TREM2^+^ TAMs are of high possibility to stimulate CD8^+^ Tex with extracellular signals.

To evaluate the key ligands secreted by TREM2^+^ TAMs, we first performed a horizontal comparison among major signalling pathways between TREM2^+^ TAMs and CD8^+^ Tex. (Figure 6F). By comparing both the relative strength and overall signalling intensity, three ligand‐receptor pairs emerged as the predominant mediators of the crosstalk between TREM2^+^ TAMs and CD8^+^ Tex: first, SPP1 was specifically secreted by TREM2^+^ TAMs and bound to CD44 on CD8^+^ Tex (Figure 6G). Second, the LGALS9–HAVCR2 interaction was observed as a part of GALECTIN signalling network (Figure 6H). Third, although with a lower probability, communication through the ALCAM–CD6 pathway was also detected (Figure 6I). Among the three ligands, we found that SPP1 was specifically expressed by TREM2^+^ TAMs among all TAM subsets (Figure 6J). Moreover, SPP1 and TREM2 were co‐expressed in TREM2^+^ TAMs (Figure 6K), which was confirmed by flow cytometry (Figure 6L,M). Cellularly, increased infiltration of TREM2^+^ TAMs was associated with elevated SPP1 expression, and SPP1 was positively correlated with the proportion of CD8^+^ Tex (Figure 6N). Molecularly, SPP1 expression was strongly correlated with ETV5 expression, whereas CD44 expression was strongly correlated with BHLHE40 expression (Figure 6O). Consistently, deconvolution analysis of microarray dataset validated that high SPP1 expression in TAMs was accompanied by increased CD8^+^ Tex (Figure 6P). Collectively, these results indicate that SPP1–CD44 axis may mediate the effect of TREM2^+^ TAMs to promote CD8^+^ T cell exhaustion.

**FIGURE 7:**
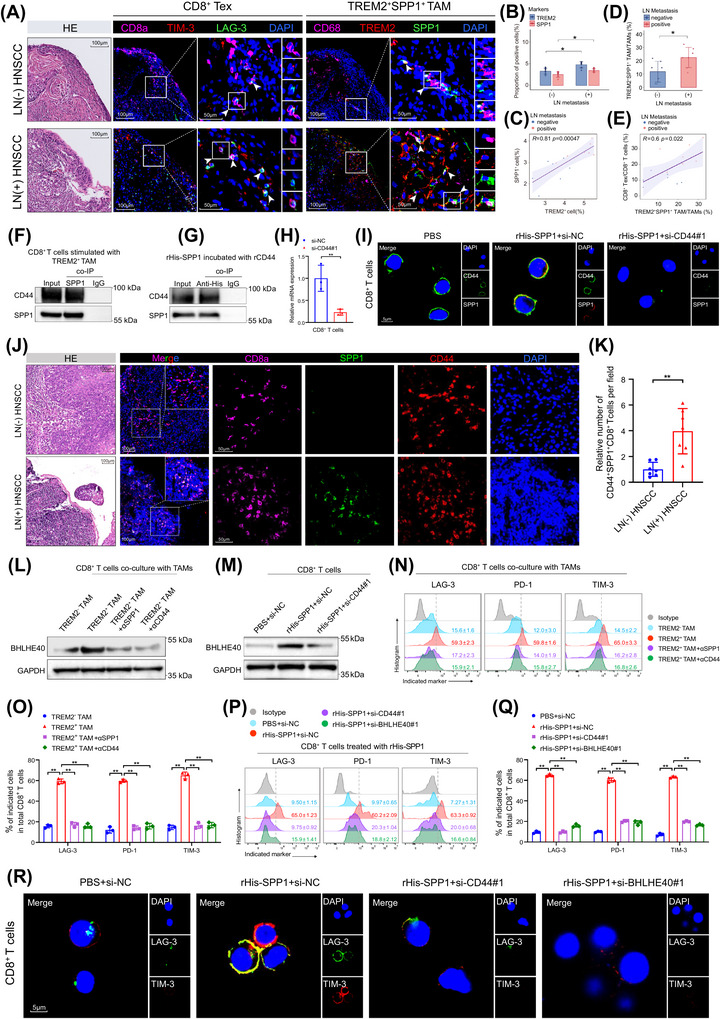
TREM2^+^ TAMs promote CD8^+^ T cell exhaustion through SPP1–CD44–BHLHE axis. (A) Representative mIHC images showing co‐infiltration of CD8^+^ Tex and SPP1^+^TREM2^+^ TAMs in LN metastasis‐positive and ‐negative tumour tissues. Scale bar, 100 µm; insets, 50 µm. (B) Expression percentage of TREM2 and SPP1 in LN metastasis‐positive and ‐negative tumours in mIHC, ^*^ *p* < .05 by Two‐way ANOVA test. (C) Correlation of expression percentage between TREM2 and SPP1 in mIHC by Spearman test. (D) Proportion of TREM2^+^SPP1^+^ TAM in total TAMs in LN metastasis‐positive and ‐negative tumours in mIHC, ^*^p < .05 by Two‐way ANOVA test. (E) Correlation of TREM2^+^SPP1^+^ TAM and CD8^+^ Tex infiltration proportion in mIHC by Spearman test. (F) co‐IP followed by Western blotting analysis demonstrating the interaction between SPP1 and CD44 in CD8^+^ T cells co‐cultured with TREM2^+^ TAMs. (G) Western blotting analysis for the binding of recombinant CD44 and His‐tagged SPP1 in co‐IP assay. (H) qRT–PCR result showing relative CD44 mRNA expression in CD8^+^ T cells transfected with si‐NC or si‐CD44. Error bars show the mean ± SD, ^**^ *p* < .01 by Two‐way ANOVA test. (I) Immunofluorescence showing the co‐localisation of SPP1 and CD44 on CD8^+^ T cells with or without CD44 silencing. Scale bar: 5 µm. (J) Representative mIHC images of SPP1 and CD44 co‐location on CD8^+^ T cell membranes in LN metastasis‐positive and ‐negative tumour tissues. Scale bar: 100 µm; inset scale bar: 50 µm. (K) Quantification of CD44^+^SPP1^+^CD8^+^ T cells across LN metastasis‐positive (*n* = 7) and ‐negative (*n* = 7) tumours by mIHC. Error bars show the mean ± SD, ^**^ *p* < .01 by Two‐way ANOVA test. (L) Western blotting analysis of BHLHE40 expression in CD8^+^ T cells co‐cultured with TAM subsets with or without anti‐SPP1 or anti‐CD44 neutralising antibodies. (M) Western blotting analysis of BHLHE40 expression in CD8^+^ T cells stimulated with rSPP1, with or without CD44 knockdown. (N) and (O) Flow cytometric analysis of exhaustion markers on CD8^+^ T cells co‐cultured with TAM subsets with or without anti‐SPP1 or anti‐CD44 antibodies. Error bars show the mean ± SD, ^**^ *p* < .01 by Two‐way ANOVA test. (P) and (Q) Flow cytometric analysis showing exhaustion markers on CD8^+^ T cells treated with rSPP1, with or without CD44 or BHLHE40 knockdown. Error bars show the mean ± SD, ^**^ *p* < .01 by Two‐way ANOVA test. (R) Immunofluorescence showing the expression of exhaustion markers on CD8^+^ T cells treated with rSPP1, with or without CD44 or BHLHE40 knockdown. Scale bar: 5 µm. ANOVA, Analysis of Variance; SD, standard deviation; si‐NC, small interfering RNA negative control; TAMs, tumour‐associated macrophages; Tex, exhausted T cells.

To confirm that SPP1–CD44 axis mediates the CD8^+^ T cell exhaustion induced by TREM2^+^ TAMs, we first examined SPP1 expression in TREM2^+^ TAMs in HNSCC tumour tissues using mIHC. The results showed that SPP1 and TREM2 were highly co‐localised, and their co‐expression was increased in LN metastatic HNSCC (Figure 7A–D). Moreover, the abundance of SPP1^+^TREM2^+^ TAMs was positively associated with the infiltration level of CD8^+^ Tex (Figure 7A,E). These results suggest that SPP1 derived from TREM2^+^ TAMs is highly correlated with CD8^+^ T cell exhaustion and LN metastasis. We then validated whether TREM2^+^ TAMs induced the CD8^+^ T cell exhaustion through SPP1–CD44 axis. Co‐immunoprecipitation (co‐IP) assay was conducted to demonstrate a direct interaction between SPP1 and CD44 (Figure 7F,G). Immunofluorescence staining confirmed that SPP1 co‐localised with CD44 on the plasma membrane of CD8^+^ T cells, which was abolished upon CD44 silencing, indicating that SPP1 binds specifically to CD44 on CD8^+^ T cells (Figure 7H,I). The interaction between SPP1 and CD44 on CD8^+^ T cells was also confirmed in HNSCC tissues via mIHC analysis, which revealed a significantly higher level of SPP1 and CD44 co‐localisation on CD8^+^ T cells in LN metastasis‐positive HNSCC tissues than in LN metastasis‐negative tissues (Figure 7J,K). Subsequently, the in vitro experiments were performed, and we found that blocking SPP1 or CD44 significantly impaired the ability of TREM2^+^ TAMs to upregulate the expression of BHLHE40 and exhaustion markers in CD8^+^ T cells (Figure 7L–O). Moreover, silencing CD44 or BHLHE40 attenuated the directly promoted effect of SPP1 in inducing the expression of exhaustion markers in CD8^+^ T cells (Figure 7P–R). Taken together, these findings indicate that TREM2^+^ TAMs promote CD8^+^ T cell exhaustion by activating BHLHE40 through the SPP1–CD44 axis, thereby facilitating immune evasion and LN metastasis in HNSCC.

### 3.7 Construction of an HNSCC prognostic model based on TREM2^+^ TAMs

**FIGURE 8:**
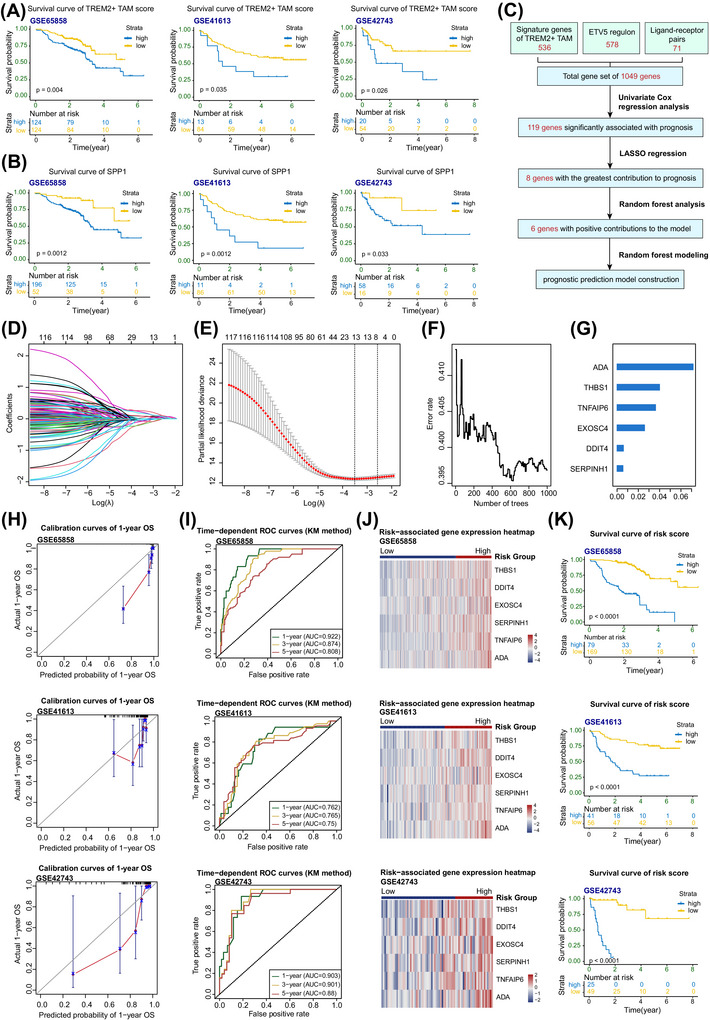
Construction of an HNSCC prognostic model based on TREM2^+^ TAMs. (A) Kaplan–Meier survival curves based on TREM2^+^ TAM infiltration scores from three microarray datasets. The samples were stratified into high‐ and low‐score groups using the optimal cutoff by log‐rank test. (B) Kaplan–Meier survival curves based on SPP1 expression levels from three microarray datasets. The samples were divided into high‐ and low‐expression groups using the optimal cutoff, by log‐rank test. (C) Workflow diagram of prognostic model construction. (D) LASSO coefficient profiles of 119 genes in the training set (GSE65858). (E) Ten‐fold cross‐validation identifying the optimal value of the penalty parameter (λ). (F) Relationship between error rate and the number of classification trees in RSF model. (G) Importance scores of the top 6 genes selected by the RSF model. (H) Calibration curves of 1‐year OS predictions in training set and validation sets. (I) Time‐dependent ROC curves and AUC values for 1‐, 3‐, and 5‐year OS predictions in both training and validation sets, using the Kaplan–Meier Method. (J) Heatmap of risk‐associated gene expression in training and validation sets. The samples were stratified into high‐ and low‐risk groups using the Youden index, by log‐rank test. (K) Kaplan–Meier survival curves based on risk scores in training and validation sets. The samples were divided into high‐ and low‐risk groups using the Youden cutoff, by log‐rank test. AUC, Area under the curve; HNSCC, head and neck squamous cell carcinoma; LASSO, least absolute shrinkage and selection operator; OS, overall survival; RSF, Random Survival Forest; TAMs, tumour‐associated macrophages.

Given the critical role of TREM2^+^ TAM infiltration and SPP1 secretion in promoting CD8^+^ Tex formation and subsequent LN metastasis in HNSCC, we first performed survival analysis of the basis of the TREM2^+^ TAM infiltration scores as well as SPP1 expression levels in three independent microarray datasets. The results revealed that HNSCC patients with higher TREM2^+^ TAM infiltration or elevated SPP1 expression exhibit significantly worse survival outcomes (Figure 8A,B), confirming the strong association between the TREM2^+^ TAMs and poor prognosis.

To evaluate the prognostic significance of TREM2^+^ TAMs, we developed a model to predict patient outcomes in HNSCC patients on the basis of TREM2^+^ TAMs. The workflow diagram illustrates the construction process of the HNSCC prognostic model (Figure 8C). For model development, microarray datasets were divided into one training set (GSE65858) and two validation sets (GSE41613 and GSE42743). A primary gene set consisting of 1049 genes was generated by intersecting the signature genes of TREM2^+^ TAMs (536 genes) (Table S1), the ETV5 regulon (578 genes) (Table S2), and genes involved in ligand–receptor interactions with CD8^+^ Tex (71 genes) (Table S3). In the first step, univariate Cox regression analysis was performed on the training set, identifying 119 genes associated with prognosis. We sequentially applied LASSO regression to these 119 genes. The regularisation parameter was selected under *λ.1se* to obtain a parsimonious model, resulting in 8 genes with the greatest prognostic contribution (Figure 8D,E). To further refine the model, we employed an RSF algorithm. Six key genes (THBS1, TNFAIP6, and DDIT4 from TREM2^+^ TAM signature genes, and ADA, EXOSC4, and SERPINH1 from ETV5 regulon) were selected by RSF, and the final prognostic model was trained on them (Figure 8F,G). To evaluate the effectiveness of the prognostic model, we assessed its performance in both the training and validation cohorts. Calibration curves and time‐dependent receiver operating characteristic (ROC) analyses were preliminarily applied to compare the predicted survival probabilities with the real observed values (Figure 8H,I). The model achieved high AUC values for predicting 1‐, 3‐, and 5‐year OS in the training set (.922,.874, and.808), and showed robust predictive accuracy in the validation sets GSE41613 (.762,.765, and.750) and especially GSE42743 (.903,.901, and.880) (Figure 8I). To further assess the predictive capacity of the model, we utilised risk scores calculated by the model to stratify patients into high‐ and low‐risk groups on the basis of a cutoff determined by the Youden Index. A heatmap revealed that all six final genes tended to be enriched in the high‐risk group (Figure 8J). Consistently, patients in the high‐risk group exhibited significantly worse OS than those in the low‐risk group (Figure 8K). Collectively, these findings support the successful construction of a prognostic model and further highlight the association between high TREM2^+^ TAM infiltration and poor prognosis of HNSCC.

## 4 DISCUSSION

LN metastasis is the main metastatic route and an early sign of distant dissemination in HNSCC, predicting a dire prognosis.^6^, ^7^, ^8^ A critical driver of LN metastasis is the development of a suppressive TIME, manifested as impaired tumour‐reactive responses and immune cell exhaustion, which fosters immune evasion and forms a metastatic niche.^9^, ^12^ However, the mechanistic dynamics governing this transition to HNSCC LN metastasis remain poorly understood. In this study, we integrated public scRNA‐seq datasets with microarray data to map the metastatic TIME landscape and identify key contributors to HNSCC LN metastasis. We found that LN metastatic HNSCC presented a suppressive TIME characterised by high infiltration of CD8^+^ Tex. CD8^+^ Tex are well known to exhibit functional deficits, including impaired proliferation and reduced cytotoxicity.^48^, ^49^ Pseudotime analysis indicated that CD8^+^ Tex developed through a gradual trajectory, beginning with CD8^+^ Tn and progressing through the effector CD8^+^ T cell state. Importantly, we identified BHLHE40 as a key TF driving CD8^+^ Tex formation in HNSCC, consistent with a previous study which reported that BHLHE40 promotes the development of intermediate Tex.^50^ These results revealed a suppressive TIME in LN metastatic HNSCC characterised by CD8^+^ Tex formation, highlighting the importance of identifying upstream regulators responsible for this transition.

Intercellular crosstalk plays a central role in regulating cell polarisation and altering biological behaviour.^51^ In this study, we found that among all the components in HNSCC TIME, TREM2^+^ TAMs represented the strongest association with elevated CD8^+^ Tex infiltration. TREM2^+^ TAMs have been identified as a terminally differentiated subset,^52^, ^53^ and were widely depicted as an immunosuppressive phenotype in various human cancers.^54^, ^55^, ^56^, ^57^ Consistently, we found that TREM2^+^ TAMs represented an ETV5‐driven terminally differentiated state and promoted CD8^+^ T cell exhaustion in HNSCC, thereby facilitating LN metastasis and contributing to worse clinical outcomes. Mechanistically, TREM2^+^ TAMs promote CD8^+^ T cell exhaustion by upregulating BHLHE40 in CD8^+^ T cells via SPP1–CD44 signalling axis. SPP1 has been extensively documented to co‐express with TREM2 in TAMs, which further supports our definition of TREM2^+^ TAMs.^58^, ^59^ These results enhanced our understanding of the role of TREM2^+^ TAMs in shaping the TIME, highlighting their critical role in driving LN metastasis in HNSCC.

Clinically, given the truth that insidious LN metastasis hinders the early diagnosis of high‐risk HNSCC patients,^60^, ^61^, ^62^ we utilised TREM2^+^ TAMs‐related genes to develop a novel prognostic model. The combination of RSF method markedly enhanced the predictive accuracy of our clinical prediction model, compared with applying only COX or LASSO.^63^, ^64^ In particular, the Youden index was used to define the optimal high/low‐risk threshold in ROC curves, maximising discriminative power for clinicians’ decision‐making.^65^ We ultimately constructed a prognostic model for HNSCC based on six representative genes of TREM2^+^ TAMs. For instance, SERPINH1 was reported to be overexpressed in HNSCC, acting as a prognostic biomarker.^66^, ^67^ Taken together, these findings underscore the prognostic value of TREM2^+^ TAMs and reinforced our findings on their correlation with poor prognosis in HNSCC.

While our study provides a comprehensive view, limitations exist. The interplay between the TIME and malignant epithelial cells remains insufficiently characterised. The intercellular pathways through which the SPP1–CD44 axis drives CD8^+^ T cell exhaustion merit further in‐depth functional studies. Furthermore, generalising our model to other cancers will demonstrate its universality and robustness.

## 5 CONCLUSIONS

Overall, our study integrated scRNA‐seq, microarray data, and a clinical patient cohort to reveal an immunosuppressive TIME in LN metastatic HNSCC characterised by high infiltration of CD8^+^ Tex. We identified TREM2^+^ TAMs as key contributors to LN metastasis in HNSCC by driving CD8^+^ Tex formation via the SPP1–CD44–BHLHE40 axis. Leveraging these findings, we constructed a prognostic model for HNSCC. Our work deepens the understanding of cancer biology and identifies TREM2^+^ TAMs as potential therapeutic targets for HNSCC treatment.

## AUTHOR CONTRIBUTIONS

Zhuokai Wu, Chixing Cheng, Zhaoxin Li, Minyi Ren, Hongxi Cao, Hanhao Zheng, and Yixi Wang designed the study. Zhuokai Wu mainly carried out the bioinformatics analysis. Chixing Cheng and Hanhao Zheng designed and performed the in vitro experiment. Weijie Huang, Jun Wang, Sien Zhang and Yixi Wang provided the clinical samples and performed the clinical data analyses. Zhuokai Wu, Chixing Cheng, Minyi Ren, Hongxi Cao, Lixian Wu, Tingyi Lee, and Yixi Wang carried out the experiments and wrote the manuscript. Hongxi Cao, Weijie Huang, Jun Wang, and Hanhao Zheng made critical revisions to the manuscript. All authors read and approved the final version of the manuscript.

## CONFLICT OF INTEREST STATEMENT

The authors declare no conflicts of interest.

## ETHICS STATEMENT

The patient‐derived tumour tissues and peripheral blood included in our study were performed in strict accordance with international standards and the sampling guidelines of the Ethics Committee of Guanghua Hospital of Stomatology (Guangzhou, China), affiliated with Sun Yat‐sen University, with Institutional Review Board Approval (KQEC‐2025‐065‐01).

## Supporting information



Supporting Information

## Data Availability

The scRNA‐seq datasets analysed in the present study are available in the GEO database (accession codes: GSE164690,^20^ GSE139324,^21^ GSE226620,^22^ GSE234933,^23^ GSE182227,^24^ and GSE181919^25^), https://www.ncbi.nlm.nih.gov/geo/, and the HCA database via CZ CELLxGENE, https://cellxgene.cziscience.com/collections/3c34e6f1‐6827‐47dd‐8e19‐9edcd461893f.^26^ Microarray data were retrieved from the GEO database (accession codes: GSE65858,^27^ GSE41613,^28^ and GSE42743^28^). Data generated in this study are provided within the article and its supplementary files, and are available from the corresponding author upon reasonable request.
