# Supplementary material for: Comprehensive tumour‐immune profiling reveals TREM2+ tumour‐associated macrophages facilitating lymph node metastasis in head and neck squamous cell carcinoma
Source: Clin Transl Med. 2026 Jan 30;16(2):e70604. doi: 10.1002/ctm2.70604 (PMC12856236; doi:10.1002/ctm2.70604)
Supplement: Supplementary file 1 — Supporting Information [file CTM2-16-e70604-s001.pdf]

# Comprehensive tumor-immune profiling reveals TREM2$^+$ tumor associated macrophages facilitating lymph node metastasis in head and neck squamous cell carcinoma

**Short running title:** TREM2$^+$ TAMs promote HNSCC LN metastasis

Zhuokai Wu[1#], Chixing Cheng[2,4#], Zhaoxin Li[1], Minyi Ren[1], Hongxi Cao[1], Weijie Huang[1], Jun Wang[1], Lixian Wu[1], Tingyi Lee[1], Sien Zhang[1*], Hanhao Zheng[3,4*], Yixi Wang[1*]

1. Hospital of Stomatology, Guanghua School of Stomatology, Guangdong Provincial Key Laboratory of Stomatology, Sun Yat-sen University, Guangzhou, China

2. Department of Urology, The Fifth Affiliated Hospital, Sun Yat-sen University, Zhuhai, Guangdong, P. R. China

3. Department of Urology, Sun Yat-sen Memorial Hospital, Sun Yat-sen University, Guangzhou, Guangdong, P.R. China

4. Guangdong Provincial Key Laboratory of Malignant Tumor Epigenetics and Gene Regulation, Sun Yat-sen Memorial Hospital, State Key Laboratory of Oncology in South China, Guangzhou, Guangdong, P.R. China

***Correspondence Authors:**

Sien Zhang: zhangsen5@mail.sysu.edu.cn

Hanhao Zheng: zhenghh27@mail.sysu.edu.cn

Yixi Wang: wangyx263@mail.sysu.edu.cn

Mailing address: No.56, Lingyuan West Road, Yuexiu District, Guangzhou,China 510000

Tel: +86-020-83702080

**#These authors contributed equally to this study.**

# Supplementary Figures

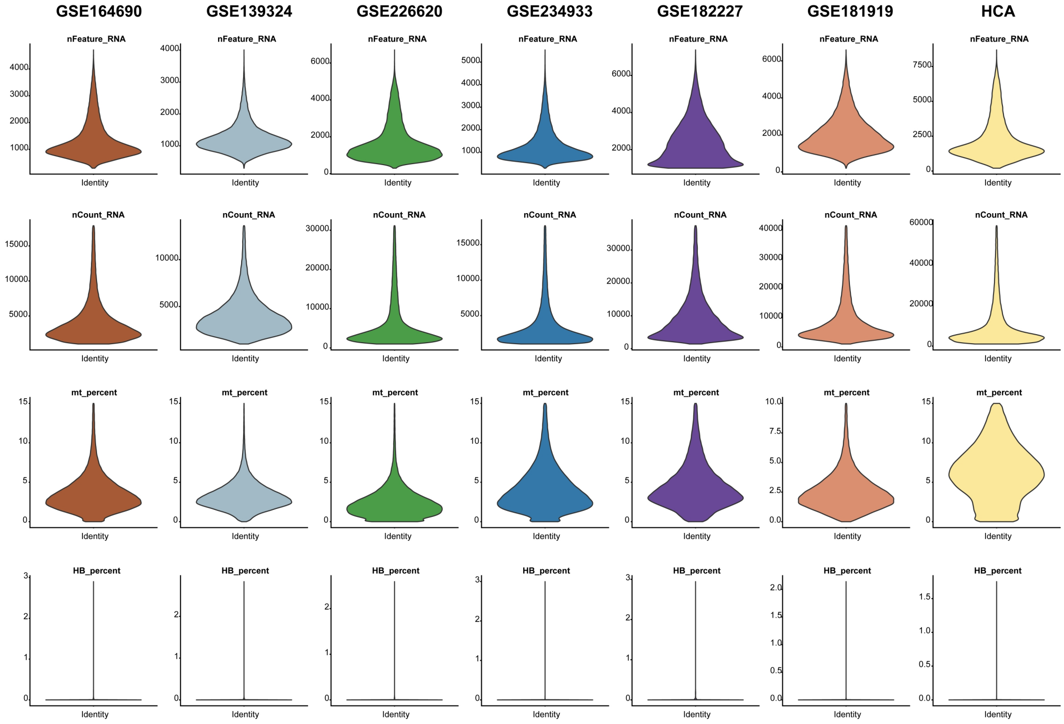


**Figure S1. Supplement data for single-cell quality control.**

Number of detected genes, total unique molecular identifier (UMI) counts, mitochondrial and hemoglobin gene reads for each dataset after quality control.

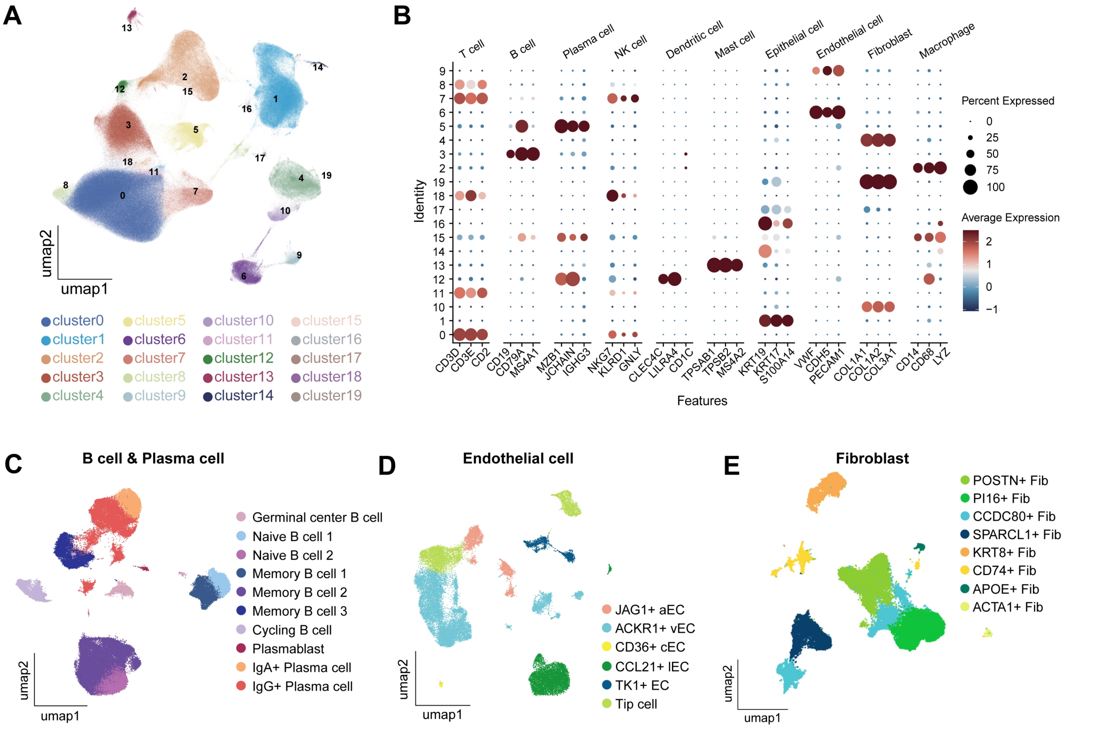


**Figure S2. Supplement data for single-cell cell types annotation.**

**A** Uniform Manifold Approximation and Projection (UMAP) plot showing Primary cell clusters.

**B** Dot plot showing the expression of marker genes across 20 primary clusters.

**C** UMAP plot of B cell and plasma cell subsets colored by identified subsets.

**D** UMAP plot of endothelial cell subsets colored by identified subsets. EC, endothelial cell; aEC, arteries EC; vEC, veins EC; cEC, capillaries EC; lEC, lymphatic EC.

**E** UMAP plot of fibroblast subsets colored by identified subsets. Fib, Fibroblast.

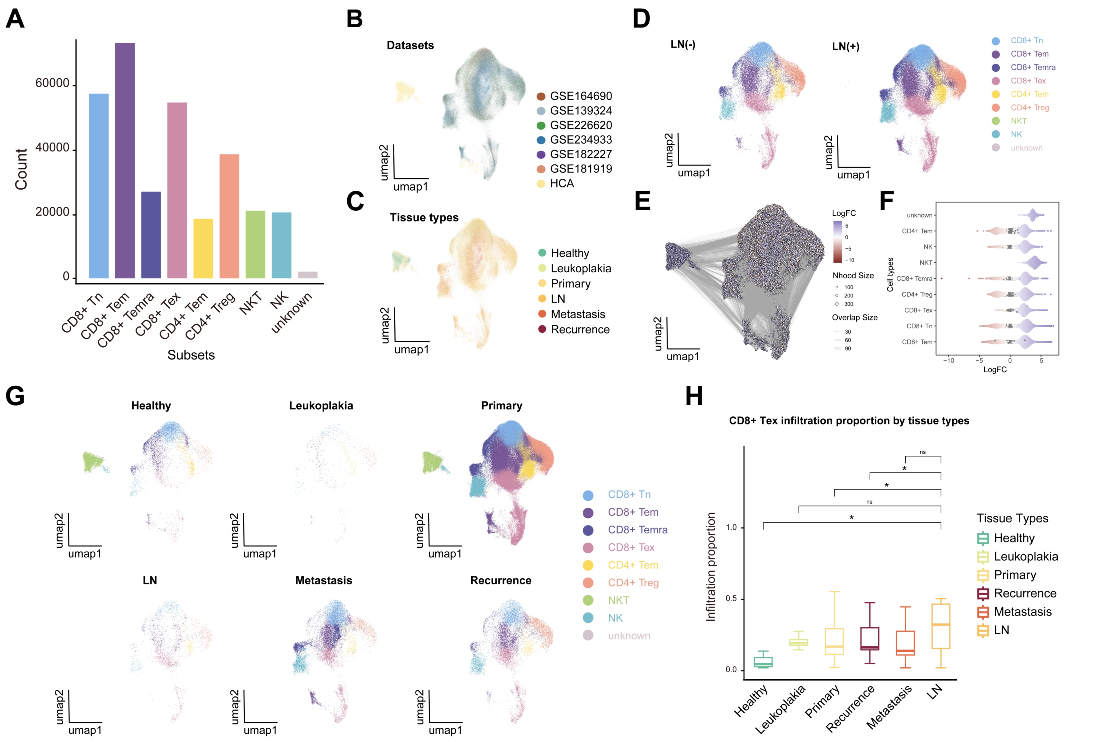


**Figure S3. Supplementary data showing the correlation between CD8$^{+}$ Tex and LN metastasis.**

**A** Total number of each subsets of T cells.

**B** UMAP plot displaying subsets colored by original datasets after batch effect correction.

**C** UMAP plot showing the distribution of subsets from different tissue types.

**D** UMAP plot of T cell subsets split by lymph node (LN) metastasis states (positive and negative).

**E** UMAP plot of T cell neighborhoods identified by MiloR, colored by log fold change (logFC) between LN metastasis-positive and -negative samples.

**F** Average logFC of neighborhoods identified by MiloR within each T cell subset.

**G** UMAP plot of T cell subsets split by tissue types.

**H** Infiltration percentage of CD8$^{+}$ Tex across tissue types, ns nonsignificant, *$p < 0.05$ by Mann-Whitney U test.

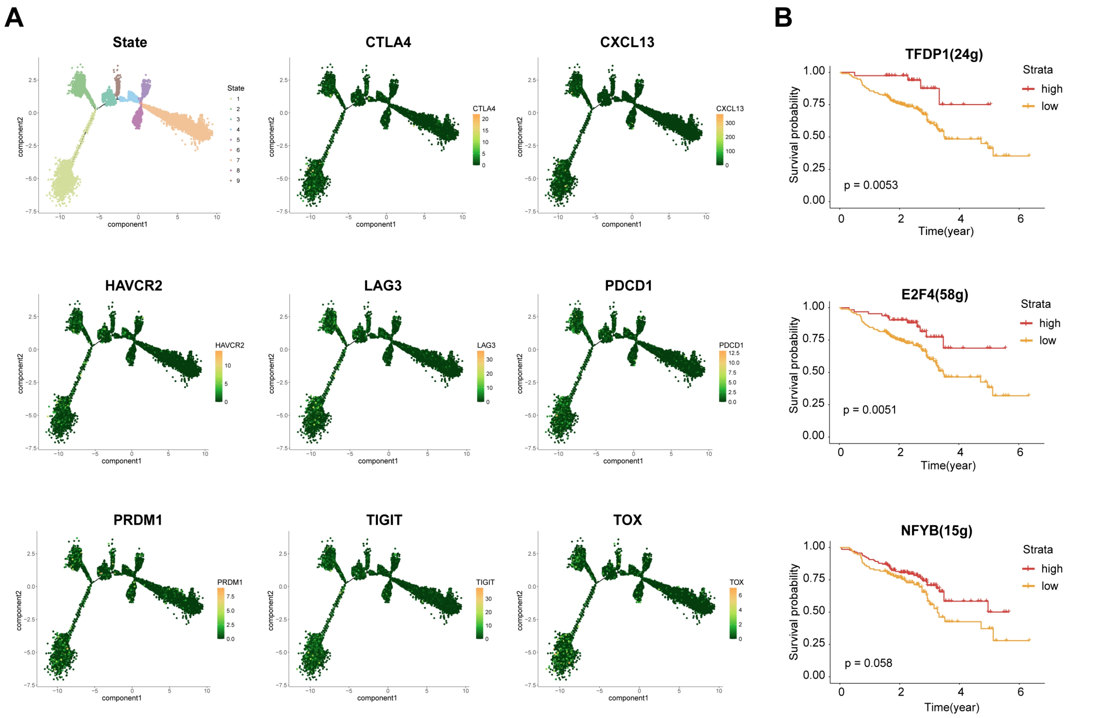


**Figure S4. Supplementary data showing exhaustion-related transcriptional profiles of CD8⁺ Tex.**

**A** Differentiation trajectories of $CD8^+$ T cells colored by state (top left), others show the distribution of marker genes of $CD8^+$ exhausted T cells ($CD8^+$ Tex).

**B** Kaplan–Meier survival curves based on TFDP1, E2F4 and NFYB regulon scores in HNSCC patients. Samples were stratified into high- and low-score groups (optimal cutoff), by log-rank test.

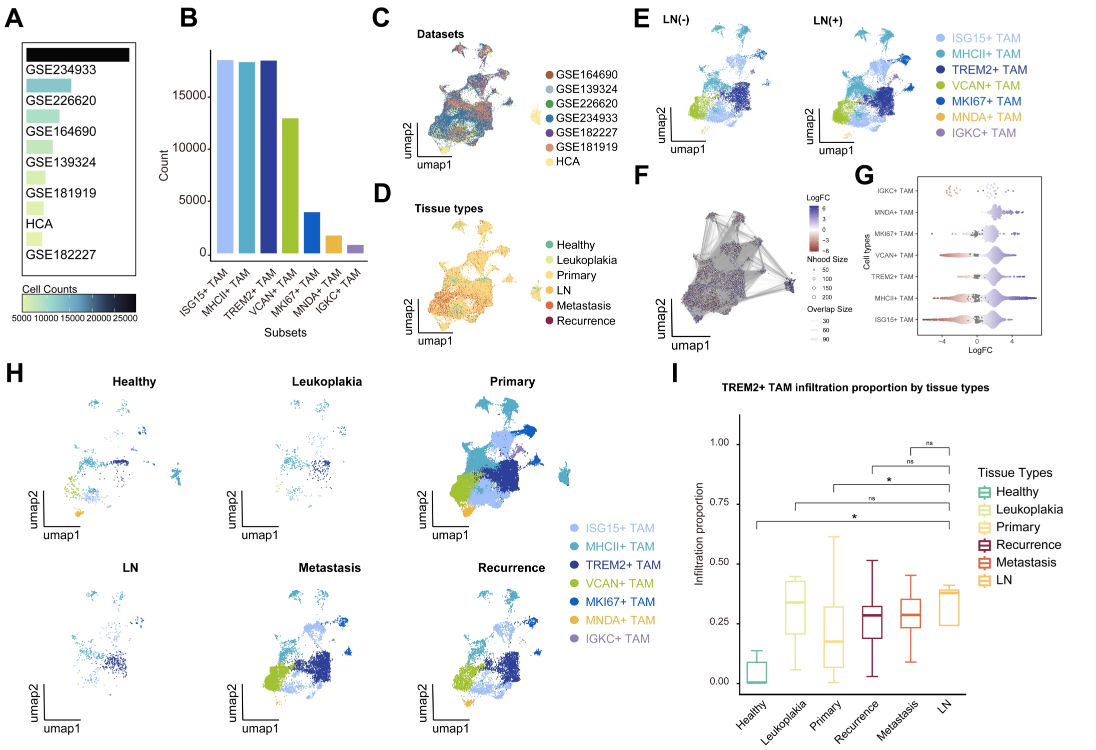


**Figure S5. Supplementary data showing the correlation between TREM2$^{+}$ TAMs and LN metastasis.**

**A** Bar plot showing the number of subsets contributed by each dataset.

**B** Total number of each subset of tumor-associated macrophages (TAMs).

**C** UMAP plot displaying subsets colored by original datasets after batch effect correction.

**D** UMAP plot showing the distribution of subsets from different tissue types.

**E** UMAP plot of TAM subsets split by LN metastasis state (positive and negative).

**F** UMAP plot of TAM neighborhoods identified by MiloR, colored by logFC between LN metastasis-positive and -negative samples.

**G** Average logFC of neighborhoods identified by MiloR within each TAM subsets.

**H** UMAP plot of TAM subsets split by tissue types.

**I** Infiltration percentage of TREM2$^{+}$ TAMs in tissue types, ns nonsignificant, *$p < 0.05$ by Mann-Whitney U test.

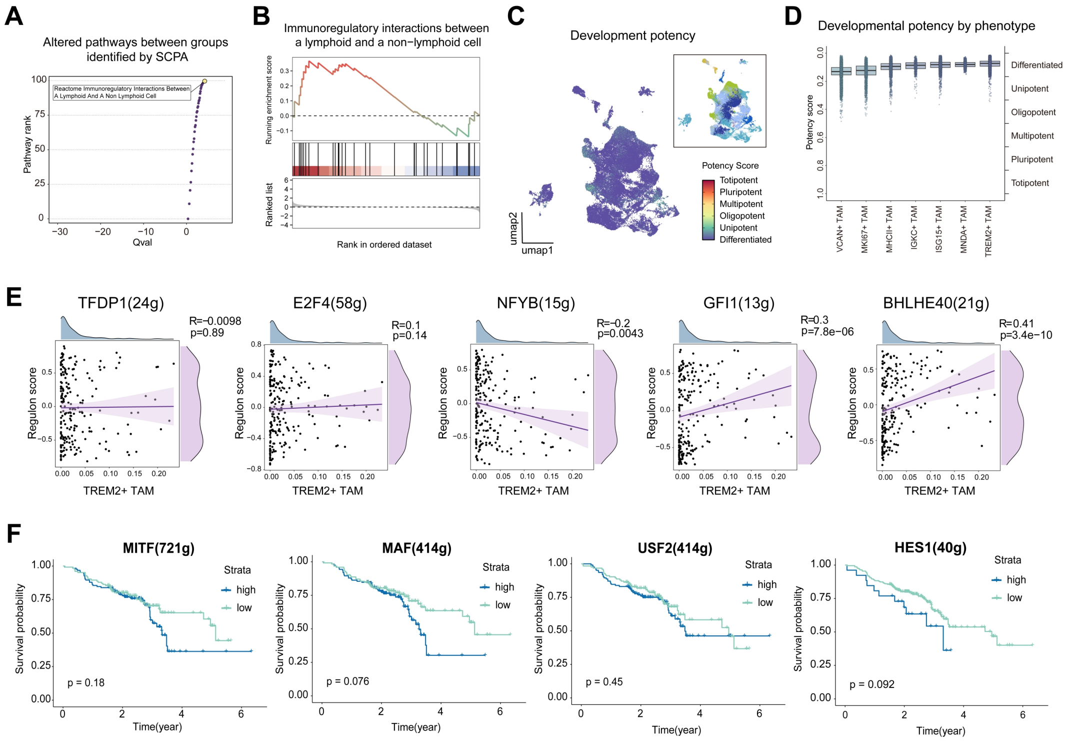


**Figure S6. Supplementary data showing transcriptional and phenotypic features of TREM2⁺ TAMs.**

**A** Single-Cell Pathway Analysis (SCPA) identifying alternated pathway activity in LN metastasis–positive group compared to -negative groups, highlighting the “Immunoregulatory Interaction Between a Lymphoid and a Non-lymphoid Cell” pathway.

**B** Gene Set Enrichment Analysis (GSEA) showing increased activity of the “Immunoregulatory Interaction Between a Lymphoid and a Non-lymphoid Cell” pathway in LN metastasis-positive group compared to -negative group.

**C** UMAP plot showing the distribution of developmental potency scores calculated by CytoTRACE2, with the inset colored by subsets.

**D** Box plot showing developmental potency score for each subset.

**E** Correlation between TREM2⁺ TAM infiltration and CD8⁺ Tex-specific transcription factor (TF) scores in scRNA-seq by Spearman test.

**F** Kaplan–Meier survival curves based on MITF, MAF, USF2 and HES1 regulon scores in HNSCC patients. Samples were stratified into high- and low-score groups (optimal cutoff), log-rank test.

# Supplementary Table

**Table S1. TREM2$^+$ TAM signature genes for prognostic model construction.**

| TREM2$^+$ TAM signature genes | | | | | |
|---|---|---|---|---|---|
| APOE | C1QC | APOC1 | C1QA | C1QB | GPNMB |
| RNASE1 | PLD3 | SLCO2B1 | DAB2 | TREM2 | PLTP |
| FCGR3A | ACP5 | CD81 | LGMN | CTSL | PLA2G7 |
| MMP14 | LIPA | MS4A4A | CD9 | A2M | TMEM176A |
| CREG1 | TMEM176B | GM2A | CD163 | CTSD | MSR1 |
| SERPING1 | NR1H3 | STAB1 | SCARB2 | NUPR1 | FPR3 |
| ACP2 | LAIR1 | TIMP2 | PMP22 | NPL | SLC40A1 |
| LILRB4 | LAMP1 | TSPAN4 | MFSD1 | ARHGAP18 | MS4A7 |
| MAFB | TTYH3 | DNASE2 | LGALS3BP | CSF1R | CTSA |
| C2 | CCL18 | CD68 | SIGLEC1 | HEXB | VAT1 |
| HEXA | MRC1 | CD14 | LHFPL2 | TCN2 | CTSC |
| CTSZ | ABHD12 | MMP9 | KCNMA1 | ASAH1 | HNMT |
| ABCA1 | GAA | SLAMF8 | SDC3 | GLMP | RNF130 |
| ADAMDEC1 | C1orf54 | TGFBI | MPP1 | FCGRT | ATP6AP1 |
| GNS | FOLR2 | IDH1 | C3AR1 | HMOX1 | MS4A6A |
| MMP12 | FUCA1 | AKR1A1 | CD84 | VSIG4 | LRP1 |
| TNS3 | NRP1 | CAPG | AKR1B1 | CMKLR1 | CD4 |
| CPM | SPP1 | FCGR1A | RAB13 | TFRC | BCAP31 |
| CTSB | IFI27 | SCPEP1 | RENBP | SPRED1 | ADAP2 |
| ATOX1 | PLAU | SLC15A3 | MGST3 | COMT | CD55 |
| ENG | SGPL1 | GPR34 | SDS | MERTK | PCBD1 |
| COLGALT1 | FABP5 | SLC31A1 | PRDX1 | TFPT | AP1B1 |
| SLC7A8 | OLFML2B | LGALS3 | IFI6 | SCD | MAF |
| GNB4 | EPHX1 | CD52 | PLXND1 | MFSD12 | GBA |
| CYFIP1 | GAL3ST4 | SMPDL3A | GCHFR | FUCA2 | HSD17B14 |
| SDSL | ADAM9 | STK17B | TMEM51 | LTB | SMIM4 |
| OTOA | CXCL9 | CD59 | FABP3 | FMNL2 | AP2A2 |
| SLC38A6 | GRN | MARCKSL1 | OLFML3 | TSC22D1 | HSD3B7 |
| FCHO2 | NEU1 | PLBD2 | PEPD | ENPP2 | ATP6V0A1 |
| GNPDA1 | CD63 | CD209 | UNC93B1 | LPAR6 | CORO1A |
| TMEM37 | FBP1 | ACE | BMP2K | CD276 | GPX3 |
| DAPK1 | PLEKHO2 | CALU | VCAN | LILRB5 | RARRES1 |
| CYTIP | JAML | RGL1 | NTAN1 | SERPINB9 | LYST |
| DUSP4 | NPC2 | MITF | FRMD4A | PLA2G15 | FAM213A |
| KLHDC8B | CST7 | LIMD2 | S100A13 | IL18BP | TRPM2 |
| SLC1A3 | BIRC3 | HLA-DOA | NCEH1 | SDC2 | FAM198B |
| PSAP | AMDHD2 | FAM20C | ABCG1 | TNFSF13 | ST14 |
| CYP27A1 | AREG | TNFSF12 | SLC25A37 | EHD1 | RHOF |
| CD72 | SH3PXD2B | CLCN7 | FAM20A | SCCPDH | ABCC3 |

| | | | | | |
|---|---|---|---|---|---|
| LINC01094 | SORBS3 | SELENOP | SCARB1 | CYB5R1 | EDEM2 |
| OSBPL1A | MINK1 | C3 | IL1B | PLEKHM2 | SLC29A1 |
| RMDN3 | ABCC5 | TCEAL3 | TGM2 | TMEM251 | SLC22A18 |
| ITGB5 | APOC2 | ARHGEF10L | SNX24 | LSP1 | VEGFB |
| SEPP1 | RAB42 | PLOD1 | CCL13 | RCAN1 | DHRS3 |
| FLVCR2 | NPC1 | CEBPA | TNFRSF21 | SLC11A2 | CD300E |
| CSTB | SLC35F6 | SLC29A3 | LACC1 | TMEM138 | CD69 |
| CNN2 | CLEC11A | CHID1 | MRAS | ME1 | SLC12A5 |
| TMEM86A | F13A1 | GADD45G | SLC2A8 | SLC37A2 | ALDH1A1 |
| BTG1 | DAPP1 | CECR1 | FN1 | SLC36A1 | ETV5 |
| WIPI1 | ADAMTSL4 | RRAGD | CHI3L1 | STK4 | FPR1 |
| TPCN1 | IFITM2 | HS3ST1 | BCL2A1 | SLC39A11 | ATP13A2 |
| SLC17A5 | PROCR | HAMP | ADORA3 | GATM | SLC38A7 |
| RBP1 | PPA1 | TMEM53 | TBC1D2 | EPHB2 | SASH1 |
| TMIGD3 | IGFBP4 | SIGLEC12 | RPL36A | S100A8 | PDK4 |
| PI4K2A | FMN1 | LPL | NAGLU | PDPN | CD163L1 |
| AC244090.1 | SERINC2 | HAGHL | GAPLINC | TIMP1 | SPARC |
| EEPD1 | IGF1 | ACVRL1 | PPIC | ECM1 | TMEM255B |
| RHOBTB3 | RPL17 | P2RY11 | PDLIM4 | SLC2A5 | SERPINB1 |
| NAMPT | RPL7 | RPL21 | RPL34 | FTL | HSBP1L1 |
| CA11 | PCK2 | ANKH | FXYD6 | ANKRD9 | HOMER3 |
| EPAS1 | PTGR1 | BHLHE41 | MAPK13 | RAC2 | AIFM2 |
| MFSD13A | CLDN7 | PMAIP1 | BLNK | ACAP1 | MRC2 |
| MATK | SPR | SREBF1 | WBP5 | GCLC | TES |
| ISG20 | ATP2B1-AS1 | SLC26A11 | PTPRO | SGPP1 | GAS6 |
| SLC2A3 | CXCR4 | SLC27A1 | LSS | WDR81 | RASSF5 |
| LINC00998 | TMEM144 | G0S2 | APPL2 | TNS1 | LXN |
| SGSH | DDIT4 | CLIP4 | EVA1B | FAM213B | TRAF1 |
| DOPEY2 | METTL9 | SAMSN1 | SMCHD1 | UNC5B | AP1S2 |
| SMIM30 | TRIP6 | IDO1 | CHCHD6 | IFITM10 | NLN |
| ANG | SELENON | RAB11FIP1 | UAP1L1 | RBMS2 | GBGT1 |
| SUCNR1 | PTGS2 | RASAL2 | TSPAN15 | GMPR | SLC23A2 |
| TMEM150A | SLC37A4 | GSTM4 | LDLRAD4 | C12orf4 | HMGB2 |
| PNPO | STK17A | B3GNT7 | TMEM63A | PDCD1LG2 | DUSP5 |
| MALT1 | MARVELD1 | ARMCX1 | S1PR2 | RUNX3 | RPP25 |
| RHBDD3 | ARPIN | TLR7 | CTNS | APOL4 | BEX3 |
| PQLC2 | IL1RN | NLRP3 | INF2 | CSTA | NFAT5 |
| ATP2B1 | PELI1 | PRKCB | WWP1 | GRAMD4 | ETV3 |
| CCDC106 | TCEAL1 | MPND | FECH | POU2F2 | LILRA5 |
| EREG | CHD1 | R3HDM4 | ADCY3 | FAM107B | ANXA6 |
| S100A9 | MGAT5 | JARID2 | MIR4435-2HG | SOCS3 | PDE4B |
| LILRA2 | AGO2 | PSIP1 | SYAP1 | TREM1 | CDC42BPB |
| KIF2A | SOX4 | CYTOR | PKIB | PTTG1 | PPIF |
| HOTAIRM1 | TRAF3IP3 | GCA | LTC4S | JOSD1 | TRIB1 |

| | | | | | |
|---|---|---|---|---|---|
| STXBP2 | CXCL8 | RYBP | RHOH | THBS1 | IVNS1ABP |
| OXSR1 | MBOAT7 | ARL5B | PHACTR1 | PHF20L1 | QPCT |
| TNFAIP6 | NKG7 | BATF | DNASE1L3 | CSF3R | BCL6 |
| LRRK2 | PLEK | AQP9 | PNPLA8 | BASP1 | LINC00152 |
| CPD | IL1R2 | C19orf38 | AC090498.1 | HLA-DQB2 | TNFRSF4 |
| IL32 | ACSL1 | OSM | CCL20 | S100B | SORL1 |
| CCL3L1 | DDX60L | | | | |

**Table S2. ETV5 regulon for prognostic model construction.**

| ETV5 regulon | | | | | |
|---|---|---|---|---|---|
| A2M | ABCC3 | ABCD3 | ABHD12 | ABI3 | ABR |
| ACOT11 | ACVRL1 | ADA | ADAM9 | ADAP2 | ADIPOR1 |
| ADORA3 | AGPAT2 | AIF1 | AIMP2 | AKR1A1 | ALOX5AP |
| AMPD3 | ANAPC11 | ANG | ANKH | ANKMY2 | ANKRD9 |
| ANKS1A | AP1B1 | AP2A2 | AP2S1 | APEX2 | APPL2 |
| AQR | ARAP1 | ARFGEF2 | ARFIP1 | ARHGAP4 | ARHGDIB |
| ARMC7 | ARPC3 | ARV1 | ASB8 | ATG4C | ATP2C1 |
| ATP7A | ATP8B4 | AXL | B3GAT3 | B3GNT7 | B4GALT7 |
| BAG5 | BANF1 | BCAS3 | BCL2L1 | BCL2L11 | BLNK |
| BMI1 | BRCA1 | BUD13 | C12orf4 | C15orf39 | C16orf70 |
| C17orf89 | C1QB | C1QC | C2 | C3AR1 | C6orf120 |
| CALHM2 | CALR | CAMK1 | CANT1 | CAPG | CAPN10 |
| CAPZB | CARD8 | CBLL1 | CCDC71 | CCDC85B | CCL2 |
| CCL23 | CCNA2 | CCR1 | CD14 | CD180 | CD200R1 |
| CD209 | CD276 | CD300A | CD36 | CD4 | CD68 |
| CD9 | CD93 | CDC16 | CDKN2C | CDKN3 | CENPB |
| CENPM | CENPP | CENPQ | CEP120 | CFLAR | CGRRF1 |
| CHEK2 | CHUK | CIZ1 | CLDN7 | CLEC11A | CLEC5A |
| CLIP2 | CLTA | CLTC | CMIP | CMKLR1 | CNDP2 |
| COG2 | COG4 | COMT | COPS7A | COX8A | CREB3L2 |
| CRYL1 | CSE1L | CSGALNACT2 | CTNND1 | CTSB | CTSD |
| CTSF | CYB561D2 | CYBB | DAB2 | DAPK1 | DCTN5 |
| DENND3 | DENND4B | DENND4C | DHRS3 | DICER1 | DLEU7 |
| DLG4 | DNAJC11 | DNAJC13 | DNAL4 | DNMT1 | DOCK4 |
| DOK3 | DUSP28 | DVL3 | EDEM2 | EEPD1 | EGR3 |
| EIF4EBP1 | EIF5A2 | ELAVL4 | ENG | EP400 | EPHB2 |
| EPN1 | ERAP1 | ERLIN2 | ETV5 | EXOSC4 | FADS1 |
| FAM102B | FAM120B | FAM129B | FAM13B | FAM175B | FAM20C |
| FAM49B | FAM96A | FARSA | FBXL17 | FBXO45 | FCER1G |
| FCF1 | FCGBP | FCGR3A | FEM1B | FEM1C | FERMT3 |
| FGD5 | FHAD1 | FKBP15 | FLT3LG | FLVCR2 | FMNL3 |
| FRMD4B | FTL | FUCA2 | G2E3 | GABPB2 | GAL3ST4 |
| GALNT6 | GAPVD1 | GBA | GFRA2 | GHRL | GIPC1 |
| GIT1 | GLRX | GMEB1 | GNPTG | GPR180 | GPR34 |
| GPR82 | GPR84 | GPX1 | GSTM1 | GZF1 | H2AFJ |
| HAAO | HAGHL | HAVCR2 | HERC4 | HEXA | HIST1H1C |
| HLA-DMA | HLTF | HMBS | HNMT | HOMER3 | HPGDS |
| HRH2 | HS3ST1 | HSBP1 | HSP90B1 | HSPA14 | HYAL2 |
| ID3 | IDH1 | IGFBP4 | IGSF21 | IGSF6 | IKBKE |
| IL1RN | IMPA1 | INPPL1 | INSIG2 | INTS1 | IPO7 |

| | | | | | |
|---|---|---|---|---|---|
| IQSEC2 | IRF5 | ITGA9 | ITGAV | ITGB5 | ITGB7 |
| ITPR2 | JAG1 | JOSD2 | KCNK13 | KCTD12 | KDELR2 |
| KIF20B | KIF3B | KIFC1 | KIFC3 | KLHDC3 | KLHDC8B |
| KRT18 | LAMB2 | LAPTM5 | LATS2 | LHFPL2 | LIG4 |
| LILRB5 | LIMK2 | LLGL2 | LPAR5 | LPCAT4 | LPXN |
| LRRC25 | LRRC8C | LRSAM1 | LTBR | LTC4S | LY86 |
| LYN | LYRM7 | LZIC | LZTR1 | MANF | MAP7 |
| 1-Mar | MCFD2 | MCOLN1 | MEA1 | MED8 | MEF2C |
| MERTK | METTL2A | MFSD3 | MGAT4A | MGAT5 | MGST3 |
| MIB2 | MICAL2 | MKNK1 | MMP14 | MMP9 | MPI |
| MRPL27 | MRPL40 | MRPL47 | MRPL52 | MS4A4A | MYL9 |
| NACC1 | NAPG | NBL1 | NCF4 | NDE1 | NDRG3 |
| NDST1 | NECAP1 | NEU1 | NEU3 | NF2 | NFE2L1 |
| NFKBIE | NFKBIZ | NINJ2 | NIP7 | NRG1 | NRP1 |
| NSUN3 | NUDT14 | NUDT16 | NUDT19 | OLFML2B | OLFML3 |
| OPA1 | OSBPL1A | OSBPL2 | OSTF1 | P4HB | PAK1IP1 |
| PAPOLG | PAQR4 | PARG | PARVB | PCK2 | PDIA6 |
| PDLIM7 | PDZD11 | PFKFB4 | PHC2 | PHKG2 | PIGF |
| PIGO | PIGS | PIK3AP1 | PIK3C2A | PKIB | PLA2G15 |
| PLA2G7 | PLEC | PLEK2 | PLEKHO2 | PLOD1 | PLOD3 |
| PLXDC2 | PLXNC1 | PLXND1 | PMP22 | PNPLA6 | POLR3H |
| PPIL1 | PRDM4 | PRPF4 | PRR13 | PSMC4 | PSMF1 |
| PTAFR | PTER | PTGFRN | PTGR1 | PTGS1 | PTPRJ |
| PTPRO | PTRH1 | PTTG1 | PUS3 | PYCARD | PYGL |
| QSER1 | QSOX1 | RAB31 | RAB3IL1 | RAC1 | RAD51AP1 |
| RAPGEF6 | RB1 | RBMS2 | RBPJ | RCBTB1 | RCBTB2 |
| RCE1 | RCSD1 | REEP4 | REPS1 | REV1 | RFC2 |
| RFC5 | RFWD3 | RHOBTB3 | RHOG | RNASE4 | RNASE6 |
| RNF185 | RNF19A | RNF214 | RPF2 | RPP25 | RTN2 |
| RUFY2 | RUNX1 | S1PR2 | SCAMP2 | SCRN3 | SDC3 |
| SDHB | SDSL | SEC24C | SEMA4B | SEMA4C | SEPT10 |
| SERINC2 | SERPINA1 | SERPINB6 | SERPINH1 | SF3B3 | SGPP1 |
| SH3RF1 | SHCBP1 | SIGLEC1 | SIGLEC10 | SIGLEC12 | SIGLEC7 |
| SIGLEC9 | SIPA1 | SIRPA | SLC12A5 | SLC16A3 | SLC1A4 |
| SLC20A2 | SLC25A30 | SLC25A40 | SLC29A1 | SLC2A9 | SLC31A1 |
| SLC35A2 | SLC35A4 | SLC36A1 | SLC37A2 | SLC38A6 | SLC39A13 |
| SLC40A1 | SLC43A3 | SLC4A1AP | SLC7A7 | SLC7A8 | SLC8A1 |
| SLC9A9 | SMARCD2 | SMC3 | SPG21 | SPIN1 | SPR |
| SPRED1 | SPRY2 | SRCAP | SRF | SSTR2 | ST14 |
| ST3GAL6 | STAB1 | STMN1 | STX16 | STXBP5 | SYK |
| SYVN1 | TAF1 | TBC1D10B | TBC1D16 | TBC1D8 | TBC1D9B |
| TCF12 | TCIRG1 | TEX264 | TFE3 | TFPT | TGFBI |
| TGFBR1 | TIMM8B | TIMP2 | TLR1 | TM2D2 | TM9SF3 |
| TMED7 | TMEM104 | TMEM106A | TMEM144 | TMEM150A | TMEM175 |

| | | | | | |
|---|---|---|---|---|---|
| TMEM192 | TMEM199 | TMEM219 | TMEM220 | TMEM37 | TMEM39A |
| TMEM51 | TMEM60 | TMEM62 | TMEM86A | TMOD2 | TNF |
| TNFAIP8L2 | TNFRSF1A | TNFSF13 | TNPO3 | TOM1 | TOP3A |
| TP53I13 | TPD52L2 | TPI1 | TPM1 | TRAPPC9 | TREM2 |
| TRIM25 | TRIM36 | TRPM2 | TSPAN14 | TSPAN31 | TTLL12 |
| TTYH3 | TWF1 | TXNDC12 | TYW1 | UBE4A | UBXN2B |
| UBXN8 | UCP2 | UGDH | UGGT1 | ULK3 | ULK4 |
| UNC5B | USP19 | UTP18 | VAMP3 | VASH1 | VCPIP1 |
| VMA21 | VPS25 | VPS37C | VPS41 | VPS52 | VPS54 |
| WDFY4 | WRB | YIF1B | YIPF2 | YIPF5 | YKT6 |
| YWHAH | ZBTB11 | ZCCHC14 | ZGPAT | ZMYND11 | ZNF200 |
| ZNF22 | ZNF26 | ZNF263 | ZNF264 | ZNF32 | ZNF410 |
| ZNF618 | ZNF677 | | | | |

**Table S3. Ligand–receptor interaction for prognostic model construction.**

| Ligand–receptor interaction | | | | | |
|---|---|---|---|---|---|
| CCL5 | CCL3 | CCL3L3 | CXCL9 | CXCL10 | CXCL13 |
| CXCL16 | MIF | IL16 | IFNG | SPP1 | ANXA1 |
| GAS6 | LGALS9 | ALCAM | APP | PTPRC | CD6 |
| CD86 | CD99 | CLEC2D | CLEC2B | CD69 | ICAM1 |
| ITGB2 | LCK | HLA-A | HLA-B | HLA-C | HLA-E |
| HLA-F | HLA-DPA1 | HLA-DPB1 | HLA-DQA1 | HLA-DMA | HLA-DMB |
| HLA-DQA2 | HLA-DOA | HLA-DQB1 | HLA-DRA | HLA-DRB1 | HLA-DRB5 |
| PECAM1 | SEMA4A | SIGLEC1 | CCR1 | CXCR3 | CXCR6 |
| CD74 | CXCR4 | CD44 | CD4 | IFNGR1 | IFNGR2 |
| ITGAV | ITGB1 | FPR1 | AXL | MERTK | HAVCR2 |
| MRC1 | CTLA4 | PILRA | KLRB1 | ITGAX | SPN |
| CD8 receptor | CD8A | CD8B | LILRB1 | PLXNB2 | |

# Supplemental Methods

## scRNA-seq data processing

Raw count matrices from each dataset were imported into R (version 4.4.1) using the R toolkit Seurat[1]. Quality control was performed individually for each dataset. Cells were retained based on the following filtering criteria: mitochondrial gene reads < 15%, hemoglobin gene reads < 3%, the number of detected genes between 200 and 10,000, and total UMI counts greater than 1,000 but below the 97th percentile. These thresholds were determined based on violin plot distributions to exclude low-quality cells and potential doublets. After quality filtering, the seven datasets were merged into one single Seurat object. The merged data underwent standard preprocessing, including normalization, identification of highly variable genes, and data scaling. During scaling, both the mitochondrial gene percentages and cell cycle scores calculated by Seurat function CellCycleScoring were regressed out to reduce technical variation. Subsequently, principal component analysis (PCA) was performed for linear dimensionality reduction, and the top 50 principal components (PCs) were retained for downstream analysis. To account for batch effects across different datasets and samples, the harmony R package was applied with sample identity as the grouping variable[2]. Harmony integration converged after five iterations.

## Reduction and clustering

Based on the integrated matrix, a shared nearest neighbor (SNN) graph was constructed using the top 20 Harmony dimensions. To investigate cellular population structure, unsupervised clustering was performed using the Louvain algorithm at

multiple resolutions. A resolution of 0.2 was selected for downstream analysis based on cluster stability evaluation using the clustree R package[3]. Subsequently, Uniform Manifold Approximation and Projection (UMAP) was applied on the same 20 Harmony dimensions for visualization of cellular heterogeneity. A total of 20 clusters were initially identified. Differentially expressed genes (DEGs) for each cluster were determined using Seurat function FindAllMarkers. Based on the expression patterns of canonical markers, along with the PanglaoDB database, 10 major cell populations were ultimately defined[4]. For subset identification, the same clustering and annotation strategy was applied.

**Differential abundance test**

Using the MiloR R framework, cells were organized into overlapping KNN-based neighborhoods to explore variations in local cell abundance[5]. By applying $\log_2$ fold change and false discovery rate thresholds, neighborhoods with statistically significant differences in abundance were identified.

**Gene Set Scoring Analysis**

Gene Set Variation Analysis (GSVA) was performed using the gsva R package to estimate variations in gene set activity at the sample level in an unsupervised manner[6]. This method transforms gene expression data into enrichment scores for predefined gene sets in each sample. The expression data used included both microarray data and pseudobulk profiles derived from scRNA-seq data. Top differentially expressed features of specific subsets or genes in TF regulons were used as input gene sets to evaluate cell infiltration levels or assess regulon activation status.

**Single Cell Pathway Analysis**

Pathway enrichment analysis was performed using the SCPA R package[7]. Single Cell Pathway Analysis (SCPA) estimates pathway-level transcriptional alterations between specific groups of cell subset through a multivariate statistical framework. SCPA returns a *q-value* metric that reflects the magnitude of change in the pathway's multivariate distribution between groups, larger *q-value* indicates higher intergroup difference.

**Proteins extraction and western blotting analysis**

For protein extraction, cells were collected and washed three times with PBS, and lysed on ice for 30 min in RIPA buffer supplemented with protease and phosphatase inhibitors. The lysates were centrifuged at 12,000 × g for 30 min at 4 °C, and the resulting supernatants were obtained for subsequent analyses. Membrane proteins were isolated using the Minut Plasma Membrane Protein Isolation and Cell Fractionation Kit (Invent Biotechnologies) assay according to the manufacturer's instructions for subsequent co-immunoprecipitation (co-IP). Protein concentrations were measured with a BCA Protein Assay Kit (CWBio). For western blotting analysis, 20 μg of total protein from each sample was resolved by 10% SDS-PAGE and then transferred onto a PVDF membrane. After blocking with goat serum to prevent nonspecific binding, the membranes were incubated overnight at 4 °C with the indicated primary antibodies, followed by incubation with HRP-conjugated secondary antibodies for 1 h at room temperature. The following antibodies were used: BHLHE40 (Proteintech, 17895-1-AP), ETV5 (Proteintech, 13011-1-AP), SPP1

(Abcam, ab214050) and CD44 (Abcam, ab316123). Protein signals were visualized using an ECL detection kit and quantified with ImageJ software.

**co-IP assays**

For protein–protein interaction analysis between SPP1 and CD44, co-IP assays were performed. $CD8^+$ T cells were collected from the lower chamber of a Transwell system after 48 h of co-culture. Approximately $1 \times 10^7$ sorted $CD8^+$ T cells were lysed using a non-denaturing lysis buffer to extract membrane-associated proteins. The lysates were incubated overnight at 4 °C with anti-SPP1 antibodies (Abcam, ab214050), followed by incubation with Protein A/G agarose beads to capture immune complexes. After extensive washing, the bound proteins were eluted and subjected to western blot analysis to detect and validate the interaction between SPP1 and CD44. Co-IP assays were also conducted using purified recombinant proteins to confirm the direct binding between SPP1 and CD44. Briefly, recombinant His-tagged SPP1 (UABIOSCIENCE) was incubated with recombinant CD44 (Gibco, Thermo Fisher Scientific) in binding buffer overnight at 4 °C. The mixture was then immunoprecipitated with anti-His antibodies or isotype control IgG, coupled with Protein A/G agarose beads. The immunocomplexes were washed and eluted for western blot analysis.

**Survival analysis**

Overall survival data were obtained from microarray datasets. GSVA was performed to calculate enrichment scores of gene sets. Then, the association between targeted gene sets, individual genes or risk scores calculated by prognostic model and

patient survival rate was evaluated. Kaplan–Meier survival curves were generated using the survival and survminer R packages to assess the prognostic significance of selected variables. Patients were stratified into high- and low-score groups based on optimal cutoff or Youden Index cutoff, differences were evaluated using log-rank test.

**Prognostic model validation**

Assessment of the prognostic model was conducted on both the training set (GSE65858) and two independent validation sets (GSE41613 and GSE42743). First, calibration curves were generated using the rms R package to evaluate the agreement between the predicted and observed survival probabilities[8]. Subsequently, time-dependent receiver operating characteristic (ROC) curve analysis was performed using the survivalROC R package to assess the discriminatory power of the model by comparing the predicted survival probabilities with the actual outcomes at different time points. To further assess the predictive capacity of the model, we used the risk score calculated by the prognostic model to stratify patients into high- and low-risk groups based on cutoff determined by the Youden Index derived from the ROC analysis[9]. Kaplan–Meier survival analysis was then applied to compare overall survival between the two risk groups, and the statistical significance was assessed using log-rank test, verifying the association between the predicted risk scores and actual survival outcomes.

**Supplementary Reference:**

1. Butler A, Hoffman P, Smibert P, Papalexi E, Satija R. Integrating single-cell transcriptomic data across different conditions, technologies, and species. *Nat Biotechnol*. 2018;36(5):411–420. doi:10.1038/nbt.4096

2. Korsunsky I, Millard N, Fan J, et al. Fast, sensitive and accurate integration of single-cell data with Harmony. *Nat Methods*. 2019;16(12):1289–1296. doi:10.1038/s41592-019-0619-0

3. Zappia L, Oshlack A. Clustering trees: a visualization for evaluating clusterings at multiple resolutions. *Gigascience*. 2018;7(7)doi:10.1093/gigascience/giy083

4. Franzén O, Gan L-M, Björkegren JLM. PanglaoDB: a web server for exploration of mouse and human single-cell RNA sequencing data. *Database (Oxford)*. 2019;2019doi:10.1093/database/baz046

5. Dann E, Henderson NC, Teichmann SA, Morgan MD, Marioni JC. Differential abundance testing on single-cell data using k-nearest neighbor graphs. *Nat Biotechnol*. 2021;40(2):245–253. doi:10.1038/s41587-021-01033-z

6. Hänzelmann S, Castelo R, Guinney J. GSVA: gene set variation analysis for microarray and RNA-seq data. *BMC Bioinformatics*. 2013;14:7. doi:10.1186/1471-2105-14-7

7. Bibby JA, Agarwal D, Freiwald T, et al. Systematic single-cell pathway analysis to characterize early T cell activation. *Cell Rep*. 2022;41(8):111697. doi:10.1016/j.celrep.2022.111697

8. Harrell F. rms: Regression Modeling Strategies R package overview. RDocumentation.org. https://hbiostat.org/R/rms/

9. Youden WJ. Index for rating diagnostic tests. *Cancer*. 1950;3(1):32–35.
